# A Genome-Wide Association Search for Type 2 Diabetes Genes in African Americans

**DOI:** 10.1371/journal.pone.0029202

**Published:** 2012-01-04

**Authors:** Nicholette D. Palmer, Caitrin W. McDonough, Pamela J. Hicks, Bong H. Roh, Maria R. Wing, S. Sandy An, Jessica M. Hester, Jessica N. Cooke, Meredith A. Bostrom, Megan E. Rudock, Matthew E. Talbert, Joshua P. Lewis, DIAGRAM Consortium, MAGIC Investigators, Assiamira Ferrara, Lingyi Lu, Julie T. Ziegler, Michele M. Sale, Jasmin Divers, Daniel Shriner, Adebowale Adeyemo, Charles N. Rotimi, Maggie C. Y. Ng, Carl D. Langefeld, Barry I. Freedman, Donald W. Bowden

**Affiliations:** 1 Department of Biochemistry, Wake Forest University School of Medicine, Winston-Salem, North Carolina, United States of America; 2 Center Genomics and Personalized Medicine Research, Wake Forest University School of Medicine, Winston-Salem, North Carolina, United States of America; 3 Center for Diabetes Research, Wake Forest University School of Medicine, Winston-Salem, North Carolina, United States of America; 4 Department of Internal Medicine, Wake Forest University School of Medicine, Winston-Salem, North Carolina, United States of America; 5 Program in Molecular Medicine and Translational Science, Wake Forest University School of Medicine, Winston-Salem, North Carolina, United States of America; 6 Department of Pediatrics, Wake Forest University School of Medicine, Winston-Salem, North Carolina, United States of America; 7 Program in Molecular Genetics and Genomics, Wake Forest University School of Medicine, Winston-Salem, North Carolina, United States of America; 8 Department of Biostatistical Sciences, Wake Forest University School of Medicine, Winston-Salem, North Carolina, United States of America; 9 Center for Public Health Genomics, University of Virginia School of Medicine, Charlottesville, Virginia, United States of America; 10 Center for Research on Genomics and Global Health, National Human Genome Center, Howard University, Bethesda, Maryland, United States of America; 11 Division of Research, Kaiser Permanente, Oakland, California, United States of America; Innsbruck Medical University, Austria

## Abstract

African Americans are disproportionately affected by type 2 diabetes (T2DM) yet few studies have examined T2DM using genome-wide association approaches in this ethnicity. The aim of this study was to identify genes associated with T2DM in the African American population. We performed a Genome Wide Association Study (GWAS) using the Affymetrix 6.0 array in 965 African-American cases with T2DM and end-stage renal disease (T2DM-ESRD) and 1029 population-based controls. The most significant SNPs (*n* = 550 independent loci) were genotyped in a replication cohort and 122 SNPs (n = 98 independent loci) were further tested through genotyping three additional validation cohorts followed by meta-analysis in all five cohorts totaling 3,132 cases and 3,317 controls. Twelve SNPs had evidence of association in the GWAS (*P*<0.0071), were directionally consistent in the Replication cohort and were associated with T2DM in subjects without nephropathy (*P*<0.05). Meta-analysis in all cases and controls revealed a single SNP reaching genome-wide significance (*P*<2.5×10^−8^). SNP rs7560163 (*P* = 7.0×10^−9^, OR (95% CI) = 0.75 (0.67–0.84)) is located intergenically between *RND3* and *RBM43*. Four additional loci (rs7542900, rs4659485, rs2722769 and rs7107217) were associated with T2DM (P<0.05) and reached more nominal levels of significance (*P*<2.5×10^−5^) in the overall analysis and may represent novel loci that contribute to T2DM. We have identified novel T2DM-susceptibility variants in the African-American population. Notably, T2DM risk was associated with the major allele and implies an interesting genetic architecture in this population. These results suggest that multiple loci underlie T2DM susceptibility in the African-American population and that these loci are distinct from those identified in other ethnic populations.

## Introduction

African Americans have a disproportionately high risk for developing type 2 diabetes (T2DM) with an estimated prevalence twice that observed for their European-American counterparts [1]. In addition to socioeconomic and behavioral risk factors, genetic factors are likely contributors to the disproportionate risk observed in this population. Genome-Wide Association Studies (GWAS) have been used extensively with great success to identify common genetic variants associated with T2DM in primarily European-derived populations [2], [3], [4]. Until recently, comparable studies have been difficult to perform in African Americans due to the greater complexity of their African-derived genome compounded by recent admixture of European-derived genes. With the development of high density SNP arrays that give reasonable coverage of the African-American genome and methods to account for admixture in this population, it has become possible to perform informative GWAS in the African-American population. The aim of this study was to identify loci that contribute to T2DM by GWAS and replication in multiple African-American samples.

## Results

### Clinical characteristics of the study samples

The clinical characteristics of the study samples used in the GWAS, Replication and Validation phases are shown in **Table 1**. The GWAS and Replication populations were similar. In both groups, the age at enrollment for the T2DM-ESRD subjects was older than for the control groups. However, the mean age at enrollment for the control groups in the GWAS and Replication phases was older than the mean age of T2DM diagnosis in the T2DM-ESRD and T2DM subjects. Notably, the use of population-based controls has not precluded the identification of bona fide associations in other efforts (e.g., [2]). All of the case groups with T2DM (T2DM-ESRD and T2DM) had a higher proportion of females; possibly reflecting the increased prevalence of T2DM among African-American women [5], participation bias and/or survival. On average, all of the groups were overweight or obese at the time of enrollment. Among case subjects, those with T2DM-ESRD had the lowest average body mass index (BMI; 29.7 kg/m^2^, **Table 1**), and the T2DM subjects without nephropathy (T2DM) had the highest average BMI (33.5 kg/m^2^, **Table 1**).

**Table 1:** Clinical Characteristics of Study Samples.

	GWAS		Replication		Validation					
					T2DM Case-Control		IRAS		IRASFS	
	T2DM-ESRD	Control	T2DM-ESRD	Control	T2DM	Control	T2DM	Control	T2DM	Control
***n***	965	1029	709	690	1246	927	115	164	97	507
**Female (%)**	61.2%	57.3%	55.7%	51.3%	64.0%	58.0%	53.9%	61.0%	70.1%	58.0%
**Age at Enrollment (years)**	61.6±10.5	49.0±11.9	60.2±10.4	48.5±12.8	57.2±11.7	46.6±13.1	56.8±8.0	54.5±8.4	53.9±11.2	40.8±13.5
**Age at T2DM diagnosis (years)**	41.6±12.4	―	39.4±12.5	―	46.1±12.6		51.1±10.7	―	51.2±11.9	―
**Age at ESRD diagnosis (years)**	58.0±10.9	―	56.7±10.9	―	―	―	―	―	―	―
**T2DM to ESRD duration (years)**	16.2±10.9	―	20.4±10.5	―	―	―	―	―	―	―
**BMI (kg/m^2^)**	29.7±7.0	30.0±7.0	29.8±6.9	29.4±7.6	33.5±7.6	30.0±7.7	32.1±6.0	29.3±5.8	34.1±6.8	29.2±6.5

Values are presented as trait mean and standard deviation.

### GWAS

**Figure 1:**
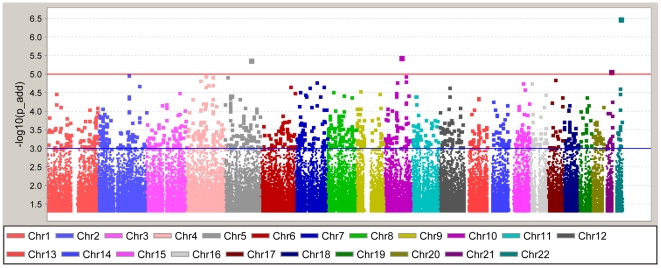
Genome-Wide Association Study Results. Results are adjusted for admixture using PC1 as a covariate in the analysis. *P*-values are shown under the additive model. The blue line at -log_10_(*P*-value) = 3 represents an additive *P*-value = 0.001 and the red line at -log_10_(*P*-value) = 5 represents a *P*-value = 1.0×10^−5^.

After the application of SNP and sample quality control metrics, 832,357 directly-genotyped, autosomal SNPs were analyzed in 965 African-American T2DM-ESRD case subjects and 1,029 African-American controls lacking T2DM and ESRD. Given the modest increase of the inflation factor with inclusion of related individuals (1.04 versus 1.06) cryptic first degree relatives were retained in the analysis. A summary of the association results is shown in **Figure 1** **and Figure S1**. The top hit was rs5750250 located on chromosome 22 in the *MYH9* (non-muscle myosin heavy chain 9) gene (*P*-value = 3.0×10^−7^, **Figure 1**). This gene has been previously associated with non-diabetic and diabetic forms of ESRD [6], [7], [8], [9]. In total, there were 126 SNPs with *P*-values<1.0×10^−4^ (**Figure 1**). In addition, we also evaluated previously identified T2DM index variants and their corresponding CEU LD blocks for association with T2DM in the African-American population (**Table S1**). Among the 37 T2DM index variants [3], [10], [11], [12], [13], [14], [15], [16], [17], [18], [19], [20], [21], [22], [23], [24], [25], [26], [27] identified to date from candidate gene studies, large scale association studies and GWAS, 35 were directly-genotyped or imputed. Among these, 20 SNPs showed consistency with the Caucasian-defined risk allele, although most were non-significant. Only rs11634397 and rs7903146 were nominally associated (*P* = 0.016 and 4.9E-05, respectively) although the direction of effect was inconsistent for rs11634397 with previous studies (OR = 0.86 with respect to the Caucasian risk allele G). Notably, additional signals of association were observed in CEU LD blocks containing the index SNP. After correction for multiple comparisons, only SNP rs4506565 in *TCF7L2* remained significant (Bonferroni-corrected P = 0.027; n = 18 SNPs (10 effective tests) contained in the CEU LD block and genotyped in the African-American GWAS). The flow of the study through the GWAS, Replication and Validation phases is outlined in **Table 2**.

**Table 2:** Study Design.

Stage	SNPs(Independent Loci)	Cases	Control	Admixture Adjustment
GWAS	832,357	T2DM-ESRD Cases (n = 965)	Population-based Controls (n = 1029)	Principal Component 1 (PC1)
Replication	712(550)	T2DM-ESRD Cases (n = 709)	Population-based Controls (n = 690)	FRAPPE (70 AIMs)
GWAS + Replication	712(550)	T2DM-ESRD Cases (n = 1,674)	Population-based Controls (n = 1,719)	
Validation	122(98)	T2DM Cases (n = 1,246)	Controls (n = 927)	FRAPPE (76 AIMs)
	122(98)	IRAS T2DM Cases (n = 115)	IRAS Controls (n = 164)	FRAPPE (70 AIMs)
	122(98)	IRASFS T2DM Cases (n = 97)	IRASFS Controls (n = 507)	FRAPPE (70 AIMs)
Validation Meta-analysis	122(98)	T2DM Cases (n = 1,458)	Controls (n = 1,598)	
Overall Meta-analysis(All 5 cohorts)	122(98)	T2DM Cases (n = 3,132)	Controls (n = 3,317)	

### Replication and GWAS + Replication Analysis of T2DM-ESRD cases and controls lacking both T2DM and ESRD

In an effort to replicate the GWAS results, the most significant 712 SNPs (n = 550 independent loci) were successfully genotyped in an additional sample of 709 African-American T2DM-ESRD cases and 690 African-American controls lacking both T2DM and ESRD (**Table 2**). In this replication analysis, 70 of the 712 SNPs (9.8%) showed nominal evidence of replication: a *P*-value<0.05 under an additive genetic model with association in the same direction. Although no SNP reached genome-wide significance (*P*-value≤2.5×10^−8^), *P*-values ranged from 7.6×10^−4^ to 6.5×10^−7^ (GWAS + Replication). The top hit from the GWAS, rs5750250, did not reach nominal significance in the replication cohort (*P*-value = 0.054).

### Validation of T2DM loci

A total of 122 SNPs were genotyped in three independent cohorts comprising a total of 1,458 African-American T2DM cases and 1,598 controls lacking both T2DM and ESRD (**Table 2**). These included 56 of the 70 SNPs with evidence of replication and 66 SNPs with more nominal evidence of significance in the combined analysis (**Table S2**). These samples allowed differentiation between association with T2DM or T2DM-ESRD while increasing power of detection for suspected T2DM loci through meta-analysis. Meta-analysis of the five putative T2DM SNPs in Validation samples, revealed association signals with *P*-values ranging from 0.011–1.8×10^−6^ (**Table S3,** **Table 3** **)**. The most significant SNP was rs7560163 (*P* = 1.8×10^−6^, odds ratio (OR) (95% confidence interval (95%CI) = 0.74 (0.63–0.87)) located intergenically between *RND3* (Ras homolog gene family, member E) and *RBM43* (RNA binding motif protein 43).

**Table 3:** GWAS + Replication, Validation and Overall *P*-values for susceptibility loci identified from the Overall meta-analysis (P<2.5×10^−5^).

Locus				GWAS			GWAS + Replication				Validation				Overall			
				T2DM-ESRD (n = 965)			T2DM-ESRD (n = 1,674)				T2DM (n = 1,458)				T2DM-ESRD + T2DM (n = 3,132)			
				Controls (n = 1029)			Controls (n = 1,719)				Controls (n = 1,598)				Controls (n = 3,317)			
SNP	Position	Alleles	Nearest Gene(s)	MAF	Additive P-Value	OR	MAF	Additive P-Value	OR	HetP-Value	MAF	Additive P-Value	OR	HetP-Value	MAF	Additive P-Value	OR	HetP-Value
						(95% CI)			(95% CI)				(95% CI)				(95% CI)	
rs7542900	Chr1:94842629	C/T	*SLC44A3* *F3*	0.46	1.4E-04	0.78	0.45	**7.7E-04**	0.85	*0.057*	0.43	**0.0024**	0.86	0.68	0.44	**6.0E-06**	0.86	0.35
						(0.69–0.89)			(0.77–0.94)				(0.78–0.96)				(0.80–0.92)	
rs4659485	Chr1:235212541	T/C	*RYR2* *MTR*	0.10	6.0E-04	0.75	0.10	**2.4E-03**	0.76	0.52	0.12	**0.0026**	0.78	0.42	0.11	**1.9E-05**	0.77	0.70
						(0.57–0.91)			(0.63–0.91)				(0.65–0.93)				(0.68–0.87)	
rs7560163	Chr2:151346182	C/G	*RBM43* *RND3*	0.13	5.6E-04	0.71	0.12	**5.5E-04**	0.77	0.22	0.15	**1.8E-06**	0.74	0.19	0.14	**7.0E-09**	0.75	0.20
						(0.58–0.86)			(0.66–0.90)				(0.63–0.87)				(0.67–0.84)	
rs3775045	Chr4:96345907	C/T	*UNC5C*	0.28	1.1E-05	1.35	0.28	**2.1E-06**	1.28	0.25	0.29	0.22	1.07	0.42	0.29	**1.7E-05**	1.17	*0.074*
						(1.18–1.55)			(1.15–1.42)				(0.95–1.20)				(1.08–1.26)	
rs6451146	Chr5:34599780	T/C	*RAI14* *-*	0.18	4.0E-05	0.70	0.17	**1.7E-05**	0.75	0.21	0.16	0.063	0.86	0.68	0.17	**1.0E-05**	0.80	0.31
						(0.58–0.83)			(0.66–0.86)				(0.74–0.99)				(0.73–0.89)	
rs6930576	Chr6:148746647	G/A	*SASH1*	0.28	1.9E-05	1.34	0.28	**7.5E-07**	1.30	0.48	0.30	0.34	1.06	0.94	0.29	**2.1E-05**	1.17	*0.093*
						(1.17–1.54)			(1.17–1.45)				(0.95–1.19)				(1.08–1.26)	
rs17103805	Chr10:86418457	A/G	*-* *FAM190B*	0.10	3.1E-04	1.43	0.10	**3.2E-06**	1.43	0.98	0.13	0.20	1.10	0.62	0.11	**1.9E-05**	1.25	0.20
						(1.18–1.73)			(1.23–1.65)				(0.95–1.29)				(1.12–1.39)	
rs2722769	Chr11:11184950	C/G	*GALNTL4* *LOC729013*	0.10	5.9E-04	0.66	0.48	**8.5E-05**	0.69	0.52	0.46	**0.0049**	0.78	0.71	0.47	**1.7E-06**	0.74	0.82
						(0.52–0.83)			(0.57–0.83)				(0.65–0.94)				(0.65–0.84)	
rs7107217	Chr11:128978900	C/A	*TMEM45B* *BARX2*	0.48	1.9E-04	0.78	0.09	**3.6E-06**	0.79	0.79	0.09	**0.011**	0.90	0.43	0.09	**3.2E-07**	0.85	0.48
						(0.69–0.89)			(0.72–0.88)				(0.81–1.00)				(0.79–0.91)	
rs1271784	Chr18:30972595	A/T	*MAPRE2*	0.22	3.6E-04	1.32	0.23	**3.6E-05**	1.28	0.64	0.27	0.080	1.08	0.51	0.25	**2.2E-05**	1.18	0.46
						(1.14–1.55)			(1.13–1.45)				(0.95–1.23)				(1.08–1.28)	

SNPs are ordered by chromosome and position (NCBI Build 36.1, hg18) with the major/minor alleles (positive strand) and corresponding gene (underlined) or nearest annotated genes (+/−500 kb). For each phase of the study, GWAS + Replication, Validation and Overall (GWAS+Replication+T2DM+IRAS+IRASFS) analyses, the minor allele frequency (MAF) in controls, additive *P*-value and odds ratio (OR) with associated 95% confidence interval (CI) with respect to the minor allele is listed.

### Meta-analysis of all African-American study samples

The association results of all 122 SNPs successfully genotyped in all five cohorts (GWAS, Replication, T2DM, IRAS and IRASFS) were used in a meta-analysis to compute an overall test of association (**Table 3**). This analysis combined results from cases (T2DM-ESRD and T2DM; *n* = 3,132) and controls (lacking both T2DM and ESRD; *n* = 3,317) for a sample size of 6,449 individuals. As a result of this analysis, one SNP reached genome-wide significance (*P*-value≤2.5×10^−8^; **Table 3** **and Figure S2**). SNP rs7560163 (*P* = 7.0×10^−9^, OR (95% CI) = 0.75 (0.67–0.84)) is located intergenically between *RND3* and *RBM43*. This SNP was tested for association with T2DM, *in silico*, by the Diabetes Genetics Replication and Meta-analysis (DIAGRAM) Consortium [3] however failed quality control filters and was not included in analysis likely due to being monomorphic as seen in a representative Caucasian population from the HapMap project (**Table S4**).

### Quantitative Trait Analysis

Exploration of putative T2DM variants with quantitative glycemic traits in a subset of African-American samples (*n* = 671 from the IRAS and IRASFS control samples, **Table S5**) revealed limited insight into the biological mechanism associated with T2DM risk. In addition, the five putative African-American T2DM susceptibility loci were tested for association with quantitative measures of glucose homeostasis in the European Caucasian population, *in silico*, by the Meta-Analyses of Glucose and Insulin-related traits Consortium (MAGIC; [16]). These results did not provide further insight into the probable role these variants may have in disease susceptibility (**Table S6**). The most significantly associated SNP in African Americans, rs7560163, failed quality controls filters and was not included in analysis likely due to being monomorphic as seen in a representative Caucasian population from the HapMap project (**Table S4**).

### Exploration of eQTLs for T2DM loci

Evaluation of three of the five putative African-American T2DM susceptibility loci for association with altered expression levels of neighboring genes revealed no strong evidence of association. However, SNP rs7542900 trended toward association with *CNN3* (β = 0.20+/−0.12, *P* = 0.095). Lack of association could be due to the small sample size (n = 90), ethnic differences (African vs. African American) or lack of identification of the causal variant. SNPs rs4659485 and rs2722769 were not evaluated as they are monomorphic in the YRI population.

## Discussion

We performed a high-density genome-wide association study to investigate the genetic determinants of T2DM in the African-American population. Meta-analysis of five study cohorts revealed a single SNP, rs7560163, near *RND3* that contributes to T2DM in the African-American population. It is noteworthy that this locus and more nominally associated loci are distinct from those implicated in previous GWAS of T2DM in primarily European-derived populations. These results are consistent with our prior observations [28], [29] that “European” genes appear to make only modest contributions to inter-individual risk of T2DM in the African-American population.

Although the associations observed reside intergenically, several neighboring genes could be implicated and have characteristics relevant to the pathophysiology of T2DM. The nearest annotated gene to SNP rs7560163, the only SNP identified to reach stringent levels of genome-wide significance in the Overall analysis (*P* = 7.0×10^−9^, OR = 0.75 (0.67–0.84); **Table 3**), is *RND3*. This gene encodes the Rho family GTPase 3 which is ubiquitously expressed and has been implicated as a regulator of actin cytoskeleton organization in response to extracellular growth factors [30], [31]. Additional SNPs that reached nominal significance in the Validation samples but failed to reach stringent criteria for genome-wide significance in the Overall analysis included SNP rs7542900 located upstream of coagulation factor III precursor (*F3*). Higher expression levels of F3 have been measured in monocytes from patients with T2DM [32] although this association could be related to unmeasured vascular complications [33], [34]. In addition, SNP rs4659485 is located intergenically between the cardiac muscle ryanodine receptor (*RYR2*) and 5-methyltetrahydrofolate-homocysteine (*MTR*) genes however, a biological relationship with T2DM is not clearly evident. SNP rs2722769 resides ∼64 kb upstream of UDP-N-acetyl-alpha-D-galactosamine polypeptide (*GALNTL4*). *GALNTL4* is a member of the large subfamily of glycosyltransferases and although little is known about its biological function, *GALNTL2* has been implicated in cholesterol metabolism in a large GWAS meta-analysis [35]. Among other top hits, rs7107217 is located downstream of BarH-like homeobox 2 (*BARX2*), a transcription factor expressed in smooth and skeletal muscle and involved in muscle differentiation [36], [37], [38].

Exploration of putative T2DM variants with quantitative glycemic traits in a subset of the samples (*n* = 671, **Table S5**) revealed limited insight into the biological mechanism associated with T2DM risk. Notably among the SNPs and traits examined, only SNP rs7107217 was nominally associated with fasting insulin (*P* = 0.011). Exploration of these variants in European Caucasian populations represented by the DIAGRAM Consortium [3] and MAGIC [16] revealed only nominal evidence of association with T2DM (rs7107217 *P* = *0.086*, located intergenically between *BARX2* and *NFRKB*; **Table S4**) and did not provide further insight into the probable role of these variants in disease susceptibility through examination of quantitative measures of glucose homeostasis (**Table S6**), respectively.

To put these findings into context, the association of *TCF7L2* with T2DM has been widely replicated across multiple ethnicities (reviewed in [39] including prior analysis of African-American samples included in this study [28], [40]). SNP rs7903146 has been the most strongly associated variant within this locus with one of the largest allelic odds ratio (OR) for a common variant, i.e. OR ∼1.35 [3]. Although rs7903146 is not typed on the Affymetrix 6.0 array and given that the genomic interval is not tagged well (max r^2^ = 0.45), only nominal evidence of association was observed in our African-American GWAS (*P* = 0.0015, rs4506565; **Table S1**). Direct genotyping of rs7903146 in the GWAS + Replication (n = 1,674 T2DM-ESRD cases and 1,719 controls lacking both T2DM and ESRD) resulted in the most strongly associated signal observed (*P* = 2.46×10^−8^) with an odds ratio (OR = 1.33, 95% CI = 1.19–1.48). This odds ratio is in the range of other signals which were observed (**Table 3**).

A notable observation common to all putative T2DM loci (**Table 3**) is the association of “protection”, i.e. and odds ratio less than 1.0, with the minor allele. Comparison with data from the International HapMap Consortium [41] confirms that the major allele in all instances is more common in the representative African samples (YRI) from Ibadan, Nigeria. This could suggest that selection for diabetogenic traits is occurring and that the more common, African-derived allele is deleterious in a more westernized environment. This is consistent with a trend we observed in prior tests of “European” T2DM associated variants in African Americans (20).

Since obesity is known to be a significant risk factor for the development of T2DM we explored the potential influence using a surrogate measure of adiposity, body mass index (BMI). As seen in **Table 1**, BMI differs significantly in the validation cohorts (*P*<0.0001). Given this significant difference, association analyses were repeated with inclusion of BMI as a covariate in the analysis. Adjustment for BMI did not substantially affect the strength of the associations observed. For example, the most significant hit from the validation analysis, rs7560163, was significantly associated with T2DM in the Validation cohorts (*n* = 1,149 T2DM cases and 919 controls with BMI data; *P* = 3.59×10^−6^) and remained the most interesting observation after adjusting for BMI (*P* = 2.83×10^−6^; data not shown). Additionally, all the case groups with T2DM (T2DM-ESRD and T2DM) had a higher proportion of females (Table 1); possibly reflecting the increased prevalence of disease in women [5]. Gender stratified analyses revealed seven of the ten most strongly associated loci were more significant in women (P = 0.0010-3.2E-07; **Table S7**) although nine of the loci remained significantly associated in men (P = 0.028-3.9E-06; **Table S7**). Notably, the power to detect association is diminished when the sample size is reduced (n = 2648 men and 3781 women).

This study has similar limitations to other GWAS conducted in minority populations. Although the current study has a modest sample size for the GWAS discovery phase compared to large-scale meta-analyses in European-derived populations, power calculations (**Tables S8 and S9**) show that this study has greater than 80% statistical power to detect effects for common variants (MAF = 0.20) consistent with published effect sizes (OR = 1.28) for T2DM (e.g. transcription factor 7-like 2 (*TCF7L2)* and potassium voltage-gated channel, KQT-like subfamily, member 1 (*KCNQ1*) with ORs 1.3–1.4; reviewed by [4]) and more modest power (<70%) to detect effects for less common variants (MAF = 0.10). The power to detect and replicate moderate level contributions to T2DM susceptibility should increase with meta-analysis of this GWAS data and other GWAS currently being conducted in African-American populations. In addition this study reports results from only directly genotyped SNPs. Effective imputation of additional SNPs would undoubtedly improve coverage of the African-American genome. While recent imputation methods development [42] show encouraging progress, rigorous empirical testing continues. A potential bias of the current study design may be that the GWAS was conducted in an African-American population of individuals with type 2 diabetes with nephropathy however; there is no specific reason why this African-American population should differ substantially from African Americans with T2DM without ESRD. For example, *TCF7L2* is strongly associated in our studies of African-American T2DM-ESRD subjects [28], [40]. In addition it should be noted that although every precaution was taken to account for population structure, as with any GWAS or candidate gene study, there may be residual population substructure. The major strength of this study is the genotyping and replication in four additional populations, thus providing support for the evidence of association observed. In addition, the study design which includes individuals with T2DM and ESRD allows for the identification of ESRD loci which are distinct from those presented herein (**Table S10**; [43]).

In conclusion, we have performed a GWAS for T2DM-ESRD in an African-American population from the southeastern United States. These results were then replicated in an additional sample recruited under identical ascertainment criteria. As a second stage of replication, a Validation study was carried out in three independent cohorts to confirm the association of suspected loci with T2DM. As a result, we have identified SNP rs7560163 that reached stringent levels of genome-wide significance and four additional loci with more nominal evidence of association. These findings require further replication in independent African-American populations as well as in additional ethnicities to confirm these findings and aid in the identification of the causal variant(s).

## Materials and Methods

### Ethics Statement

Recruitment and sample collection procedures were approved by the Institutional Review Board at Wake Forest University (GWAS, Replication, T2DM, IRAS and IRASFS samples) and Howard University (HUFS samples). Written informed consent was obtained from all study participants.

### Subjects

#### Genome-Wide Association Study (GWAS) samples and clinical characteristics

Recruitment and sample collection procedures were approved by the Institutional Review Board at Wake Forest University and informed consent was obtained from all study participants. Patients with T2DM-ESRD were recruited from dialysis facilities. T2DM was diagnosed in African Americans who reported developing T2DM after the age of 25 and who did not receive only insulin therapy since diagnosis. In addition, cases had to have at least one of the following three criteria for inclusion: a) T2DM diagnosed at least 5 years before initiating renal replacement therapy, b) background or greater diabetic retinopathy and/or c) ≥100 mg/dl proteinuria on urinalysis in the absence of other causes of nephropathy (T2DM-ESRD cases). Unrelated African-American controls without a current diagnosis of diabetes or renal disease were recruited from the community and internal medicine clinics (controls). All T2DM-ESRD cases and controls lacking T2DM and ESRD were born in North Carolina, South Carolina, Georgia, Tennessee or Virginia. DNA extraction was performed using the PureGene system (Gentra Systems; Minneapolis, MN).

#### Replication study samples and clinical characteristics

African-American T2DM-ESRD cases and controls lacking T2DM and ESRD were recruited using the same criteria as the case and control subjects that were used in the GWAS.

#### Validation study samples and clinical characteristics

*T2DM Cases*. Subjects with T2DM without evidence of nephropathy were recruited from medical clinics, churches, health fairs and community resources. Individuals were unrelated and self-described African Americans. All subjects were born in North Carolina, South Carolina, Georgia, Virginia or Tennessee. The PureGene system (Gentra Systems; Minneapolis, MN) was used for DNA extraction. *Controls*. The Howard University Family Study (HUFS) is a population-based study of African-American families enrolled from the Washington, D.C. metropolitan area. Families were not ascertained based on a given phenotype. In a second phase of recruitment, additional unrelated individuals from the same geographic area were enrolled to facilitate a nested case-control study design. A total of 1,976 samples remained after data cleaning. Diagnosis of T2DM was based on the criteria established by the American Diabetes Association Expert Committee: a fasting plasma glucose concentration ≥126 mg/dL (7.0 mmol/l) or a 2-h postload value in the oral glucose tolerance test ≥200 mg/dL (11.1 mmol/l) on more than one occasion or receiving medication for T2DM. From this sample, a subset of 927 unrelated control individuals was used for analysis. *IRAS*. The Insulin Resistance Atherosclerosis Study (IRAS) is a multicenter population-based cohort study that recruited men and women from 40 to 69 years of age living in four U.S. communities from 1992 to 1993 [44]. The study recruited approximately equal numbers of persons with normal glucose tolerance, impaired glucose tolerance and T2DM. Diabetes was defined by self-report or a fasting glucose measures >126 mg/dL at baseline or follow-up visits. The IRAS protocol was approved by local institutional review committees and all participants gave informed consent. *IRASFS*. Study design, recruitment and phenotyping for the IRAS Family Study (IRASFS) have been described [45]. Briefly, the IRASFS is a multicenter study designed to identify the genetic determinants of quantitative measures of glucose homeostasis. Members of large families of self-reported African Americans (*n* = 581 individuals in 42 pedigrees from Los Angeles, California) were recruited. Diabetes was defined by self-report, use of diabetes medications or fasting glucose measures >126 mg/dL at baseline or follow-up visits. The IRASFS protocol was approved by local institutional review committees and all participants gave informed consent.

### Genotyping and Quality Control

#### GWAS

Genotyping was performed at the Center for Inherited Disease Research (CIDR) using 1 µg of genomic DNA (diluted in 1× TE buffer and at 50 ng/µl) on the Affymetrix Genome-wide Human SNP array 6.0. DNA from cases and controls were equally interleaved on 96-well master plates to ensure technical uniformity during sample processing. To confirm sample identity, a SNP barcode (96 SNPs) was generated prior to genotyping on the Affymetrix arrays and confirmed on downstream released genotyping data. Genotypes were called using Birdseed version 2; APT 1.10.0 by grouping samples by DNA plate to determine the genotype cluster boundaries. All autosomal SNPs (n = 868,157) were included in analysis but classified on data quality with primary inference drawn from SNPs (n = 832,357) which had less than 5% missing data, Hardy-Weinberg *P*-values in cases greater than 0.0001 and in controls greater than 0.01, no significant difference in missing data rate between cases and controls and were polymorphic. The average sample call rate was 99.16% for all autosomal SNPs. Forty-six blind duplicates were included in genotyping and had a concordance rate of 99.59%. In addition, individuals whose gender call from X chromosome genotype data was discordant with the gender obtained from patient interviews were excluded from the analysis (*n* = 1). Cryptic relatedness was estimated by pairwise identity-by-descent (IBD) analysis implemented in the PLINK analysis software package (http://pngu.mgh.harvard.edu/purcell/plink/). Two duplicate samples were identified, and one sample in each duplicate pair was removed. In addition, 104 individuals were identified as cryptic first degree relatives. We also assessed heterozygosity by estimating the inbreeding coefficient using PLINK. One subject had an F value >4 standard deviations; this excess of homozygosity would suggest population substructure and this subject was removed. Our final dataset consisted of 1994 individuals in which we performed the association analysis.

#### Replication

The replication sample consisted of a population recruited under identical ascertainment criteria to that of the GWAS. A total of 749 SNPs (including 272 SNPs captured in 104 linkage disequilibrium (LD) blocks defined by an r^2^>0.50 at consecutive loci as assessed in 988 unrelated GWAS control subjects; 581 independent loci) were selected for genotyping on the Sequenom MassArray platform (Sequenom; San Diego, CA). Case and control samples were genotyped and analyzed together to avoid sample-dependent SNP calling bias. SNPs were included in analysis if genotyping was greater than 90% efficient, had a Hardy-Weinberg *P*-value≥0.001 in the replication cohort and were polymorphic (n = 712 SNPs, including 264 SNPs captured in 102 LD blocks defined by an r^2^>0.50 at consecutive loci as assessed in 988 unrelated GWAS control subjects; 550 independent loci). Forty five blind duplicate samples included in genotyping had a concordance rate of >99.9%.

#### Validation

Among the 712 SNPs genotyped during the replication phase, 122 (including 41 SNPs captured in 17 linkage disequilibrium (LD) blocks defined by an r^2^>0.50 at consecutive loci as assessed in 690 unrelated Replication control subjects; 98 independent loci) were genotyped using the iPLEX™ Sequenom MassARRAY platform (T2DM, IRAS and IRASFS) or on the Affymetrix Genome-wide Human SNP array 6.0 (Controls) for the validation phase. Genotyping was greater than 90% efficient and the 50 blind duplicate samples included in genotyping had a concordance rate of 100%.

### Analysis

#### GWAS

To address the effect of admixture in this African-American dataset we performed a Principal Components Analysis (PCA) which utilized all high quality data from the GWAS excluding regions of high LD and inversions. This approach was an iterative process whereby all high quality autosomal SNPs were used to calculate the top 50 principal components. Once calculated, the principal components were examined to determine if they were tied to regions of the genome. If so, those SNPs were excluded and the analysis repeated. The first principal component (PC1) explained the largest proportion of variation at 22% and was used as a covariate in all analyses. A direct comparison of the PCA with FRAPPE [46] analysis of 70 ancestry informative markers (AIMs; [47]) resulted in a high correlation between PC1 and the AIMs ancestry estimates, r^2^ = 0.87. The mean (SD) African ancestry proportion in 965 T2DM-ESRD cases and 1,029 controls was 0.80±0.11 and 0.78±0.11, respectively, as estimated by FRAPPE analysis. Other principal components were associated with regions of the genome, representing another unclassified source of variance. To test for association with T2DM-ESRD, genotypic tests of association were performed on each SNP individually using SNPGWA (www.phs.wfubmc.edu; [48]), an analytic package which includes the capability to perform association calculations adjusting for covariates. The primary inference was based on the additive genetic model; with note when there is strong evidence of a departure from additivity. The inflation factor was calculated as the observed mean of the chi squared statistic and compared to its theoretical expectation of 1 under the null hypothesis.

Imputation was performed for autosomes using MACH (version 1.0.16, http://www.sph.umich.edu/csg/abecasis/MaCH/) to obtain missing genotypes for previously identified T2DM index variants and to provide support for regions associated with T2DM in the African American dataset. SNPs with minor allele frequency ≥1%, call rate ≥95% and Hardy–Weinberg *P*-value ≥10^−4^ were used for imputation. A 1∶1 mixture of the HapMap II release 22 (NCBI build 36) CEU∶YRI consensus haplotypes (http://mathgen.stats.ox.ac.uk/impute/) were used as a reference panel. Imputation was performed in two steps. For the first step, 484 unrelated African-American samples were randomly selected to calculate recombination and error rate estimates. In the second step, these rates were used to impute all samples across the SNPs in the entire reference panel. Imputation results were filtered at an rsq threshold of ≥0.3 and a minor allele frequency ≥0.05.

We examined previously identified T2DM loci for association with T2DM in the African American GWAS dataset. For SNPs not available on the Affymetrix 6.0 array or from direct genotyping (n = 10), genotypes were determined from imputation. In addition to the index variant, we identified the corresponding LD block using the HapMap phase II CEU data as defined by Gabriel *et al.* [49] and implemented in Haploview. These intervals were then extracted from the African-American GWAS and the most significant SNP identified. These results were corrected for the effective number of SNPs (independent SNPs) in each locus counted using the Li and Ji method implemented in SOLAR [50]. The empirical locus-specific P-values were adjusted for multiple comparisons by Bonferroni correction for the effective number of SNPs (Table S1).

#### Replication in T2DM-ESRD cases and controls lacking T2DM and ESRD

To account for admixture in the replication cohort, ancestral allele proportions were estimated by comparing allele frequencies to 70 AIMs [47] genotyped in 44 Yoruba Nigerians and 39 European Americans. Individual ancestral proportions were generated for each subject using FRAPPE [46], an EM algorithm-based approach, under a two-population model and used as covariates in all analyses. The mean (SD) African ancestry proportion in 709 T2DM-ESRD cases and 690 controls was 0.80±0.12 and 0.76±0.13, respectively. Association analysis was performed as described for the GWAS.

#### Validation in T2DM, non-nephropathy cases and controls lacking T2DM and ESRD

In order to discriminate between association with T2DM and T2DM-ESRD, meta-analysis of three additional association analyses was performed. For the T2DM population, individual admixture proportions were estimated by comparing allele frequencies from 76 AIMs genotyped on the Sequenom MassArray (T2DM cases) or Affymetrix 6.0 array (controls) to frequencies reported in the HapMap CEU and YRI populations (unrelated samples only). Individual ancestral proportions were generated for each subject using FRAPPE [46] under a two-population model and used as covariates in all analyses. The mean (SD) African ancestry proportion in T2DM cases and controls was 0.78±0.11 and 0.76±0.12, respectively. Association analysis was performed as described for the GWAS. For the IRAS and IRASFS cohorts, ancestral allele frequencies were estimated using 70 AIMs genotyped in 44 Yoruba Nigerians and 39 European Americans. Individual ancestral proportions were generated for each subject using FRAPPE [46] under a two-population model and used as covariates in all analyses. For the IRASFS cohort, each SNP was examined for Mendelian inconsistencies using PedCheck [51]. Genotypes inconsistent with Mendelian inheritance were converted to missing. Maximum likelihood estimates of allele frequencies were computed using the largest set of unrelated African-American individuals (*n* = 58), and then genotypes were tested for departures from Hardy-Weinberg proportions. For the IRAS (unrelated individuals) and IRASFS (related individuals) cohorts, data was analyzed using a variance component measured genotype model [50]. To model T2DM as the outcome, a threshold model of the variance component measured genotype model was used. Likelihood ratio tests were computed for the tests of association with the individual SNP, modeling the correlation structure suggested by the familial relationships as appropriate, i.e. IRASFS. The family data has already been examined in detail and familial relationships corrected based on a linkage panel. *P*-values were calculated from the threshold model while the odds ratios were calculated from a logistic regression model.

#### Meta-Analyses

In order to perform GWAS + Replication, Validation (T2DM, IRAS and IRASFS) and Overall (GWAS + Replication + Validation) analyses a meta-analysis approach was taken. Meta-analysis was performed using the weighted Z-method implemented in METAL (www.sph.umich.edu/csg/abecasis/metal). This approach allows *P*-values and direction of effect to be combined independent of β-estimates, allowing for incompatibility between phenotype units as in the Fisher method [52], but with improved power and precision over Fisher's test [53]. The Z-statistic was derived from the sample-specific *P*-values and directionality of effect which were then summed with weights proportional to the square root of the sample size for each sub-study.

#### Quantitative Trait Analysis

To test for association between individual SNPs and quantitative measures of glucose homeostasis in the IRAS and IRASFS cohorts, differences in trait values by genotype were tested using the variance components model that explicitly models the correlation among related individuals as implemented in SOLAR (12). For statistical testing, trait values were transformed to best approximate the distributional assumptions of the test and to minimize heterogeneity of the variance. The primary statistical inference was the additive genetic model. All tests were computed after adjustment for age, gender, BMI and admixture adjustment.

### Exploration of eQTLS for T2DM loci

To identify potential T2DM-susceptibility genes we explored association of the putative African-American T2DM loci with transcript levels for flanking genes using gene expression profiles from the publically available HapMap Yoruba (YRI) dataset [54]. Coupling the YRI expression dataset with genotypes from the most associated loci we explored the association of SNPs with flanking genes using the variance components model and accounting for correlation among related individuals as implemented in SOLAR (12).

## Supporting Information

Figure S1 **Quartile-Quartile plot of the genome-wide association study results.**
(DOC)

Figure S2 **African-American T2DM candidate regions.** A) rs7542900 region. B) rs4659485 region. C) rs7560163 region. D) rs2722769 region. E) rs7107217 region. −log_10_ additive *P-value* from the GWAS are plotted versus position (NCBI Build 36.1, hg18). The large red diamond indicates the additive *P-value* from the GWAS of the marker(s) displayed. The large blue diamond and corresponding *P-value* indicates the additive *P-values* from the Overall analysis of the marker(s) displayed. r^2^ based on the control samples is color-coded with respect to the most significant SNP: red (0.8–1.0), orange (0.5–0.8), yellow (0.2–0.5) and white (<0.2). Gene annotations were obtained from UCSC Genome Browser (RefSeq Genes, b36). Arrows represent direction of transcription.
(DOC)

Table S1 **GWAS** ***P-values*** **for previously associated T2DM loci.** Loci are ordered by chromosome and position (NCBI Build 36.1, hg18) and referenced (Ref) by the initial publication. The African American major/minor alleles are presented on the positive strand with the Caucasian risk allele underlined. For each T2DM Index SNP, results from the African American GWAS (the minor allele frequency (MAF) for the T2DM-ESRD and control populations or combined for imputed SNPs with the corresponding additive *P-value* and odds ratio (OR) with associated 95% confidence interval (CI)) are presented with respect to the published risk allele (underlined). In addition, association results (additive *P-value* and odds ratio (OR) with associated 95% confidence interval (CI)) from recent Caucasian large-scale meta-analyses with associated references (Ref) are listed for comparison. For each index SNP, the corresponding LD block was identified using the HapMap phase II CEU data as defined by Gabriel *et al.* and implemented in Haploview. These intervals were then extracted from the African-American GWAS and the most significant SNP listed. From the GWAS, the minor allele frequency (MAF) for the T2DM-ESRD and control populations are listed with the corresponding additive *P-value* (nominal and corrected for the effective number of tests at the locus (number of SNPs genotyped in the GWAS and effective number of SNPs determined from the Li and Ji method and implemented in SOLAR)) and odds ratio (OR) with associated 95% confidence interval (CI) with respect to the African-American minor allele.
(DOC)

Table S2 **GWAS, Replication, T2DM, IRAS and IRASFS P-values for 122 GWAS SNPs genotyped on replication and validation samples.** SNPs are ordered by chromosome and position (NCBI Build 36.1) with the major/minor alleles (positive strand). For each cohort, minor allele frequency (MAF) for case and control populations are listed with the reference allele (minor allele) and corresponding additive P-value and odds ratio (OR) with associated 95% confidence interval (CI) with respect to the minor allele. Note: For IRAS-FS MAFs are derived from the overall sample including relatives. In addition, allele frequencies has been extracted from HapMap Yoruba (YRI) and CEPH (CEU) samples for comparison. Rows in red type represent the five loci which are the focus of the manuscript.
(XLS)

Table S3 **Validation** ***P-values*** **for T2DM loci across the genome.** SNPs are ordered by chromosome and position (NCBI Build 36.1, hg18) with the major/minor alleles (positive strand) and corresponding gene (underlined) or nearest annotated gene. For the T2DM, IRAS and IRASFS analyses, the minor allele frequency (MAF) for T2DM and control populations are listed with the corresponding additive *P-value*. Note: For IRASFS MAFs are derived from the overall sample including relatives. For the Validation meta-analysis the additive *P-value* and odds ratio (OR) with associated 95% confidence interval (CI) are presented with respect to the minor allele.
(DOC)

Table S4 **Association results for African-American T2DM loci in the Diabetes Genetics Replication and Meta-analysis (DIAGRAM) Consortium.** SNPs are ordered by chromosome and position (NCBI Build 36.1, hg18) and the nearest annotated gene is listed. For each SNP the major/minor alleles identified in the Overall African-American meta-analysis are indexed on the forward strand. Results from the association analysis in the Overall African-American cohort and DIAGRAM Consortium include the allele frequency (AF), odds ratio (OR) with associated 95% confidence interval (CI) and *P-value* with respect to the minor allele identified in the African-American population. SNP rs7560163 did not pass quality control filters in the DIAGRAM Consortium and was not included in analysis.
(DOC)

Table S5 **Quantitative trait meta-analysis for African-American T2DM loci across the genome.** SNPs are ordered by chromosome and position (NCBI Build 36.1, hg18) with the major/minor alleles (positive strand) and the nearest annotated gene is listed. For the IRAS and IRASFS samples, the β coefficient with respect to the minor allele is listed with the corresponding additive *P-value*. For the meta-analysis, the z-statistics is listed with the corresponding additive *P-value*.
(DOC)

Table S6 **Association results for African-American T2DM loci in the** ***Meta-Analyses*** **of Glucose and Insulin-related traits Consortium (MAGIC).** SNPs are ordered by chromosome and position (NCBI Build 36.1, hg18) with the alleles on the positive strand (African American risk alleles are underlined) and the nearest annotated gene is listed. For each SNP and trait combination, the effect size and standard error are listed with the corresponding *P-value*.
(DOC)

Table S7 **Gender stratified association analysis with T2DM.** SNPs are ordered by chromosome and position (NCBI Build 36.1, hg18) with the major/minor alleles (positive strand) and corresponding gene (underlined) or nearest annotated genes (+/−500 kb). For males and females, the additive *P*-value and odds ratio (OR) with associated 95% confidence interval (CI) with respect to the minor allele and heterozygosity P-value are listed.
(DOC)

Table S8 **Power Calculations.** Table S8a. Genome-wide association study power analysis for causal variant in complete and incomplete linkage disequilibrium with a typed variant given minor allele frequency (p) in 965 cases and 1029 controls. Table S8b. Replication power analysis for causal variant in complete and incomplete linkage disequilibrium with a typed variant given minor allele frequency (p) in 709 cases and 690 controls. Table S8c. GWAS + Replication sample power analysis for causal variant in complete and incomplete linkage disequilibrium with a typed variant given minor allele frequency (p) in 1674 cases and 1719 controls. Table S8d. T2DM power analysis for causal variant in complete and incomplete linkage disequilibrium with a typed variant given minor allele frequency (p) in 1246 cases and 927 controls. Table S8e. IRAS power analysis for causal variant in complete and incomplete linkage disequilibrium with a typed variant given minor allele frequency (p) in 115 cases and 164 controls. Table S8f. IRASFS power analysis for causal variant in complete and incomplete linkage disequilibrium with a typed variant given minor allele frequency (p) in 97 cases and 507 controls. Table S8g. Validation meta-analysis power analysis for causal variant in complete and incomplete linkage disequilibrium with a typed variant given minor allele frequency (p) in 1458 cases and 1598 controls. Table S8h. Overall power analysis for causal variant in complete and incomplete linkage disequilibrium with a typed variant given minor allele frequency (p) in 3132 cases and 3317 controls.
(DOC)

Table S9 **IRAS and IRASFS power analysis to detect a causal variant with the effect size observed in the T2DM cohort.**
(DOC)

Table S10 ***P-values*** **for putative ESRD loci across the genome.** SNPs selected from the GWAS (P<0.001) and associated in the Replication cohort (P<0.05 and directionally consistent) but which were not associated in the Validation cohort (P>0.05) and could represent putative ESRD loci. SNPs are ordered by chromosome and position (NCBI Build 36.1) with the major/minor alleles (positive strand) and corresponding gene (underlined) or nearest annotated gene. For each phase of the study, GWAS + Replication, Validation and Overall analyses, the additive *P-value* and odds ratio (OR) with associated 95% confidence interval (CI) with respect to the minor allele is listed.
(DOC)

## References

[1] Cowie CC, Rust KF, Byrd-Holt DD, Eberhardt MS, Flegal KM (2006). Prevalence of diabetes and impaired fasting glucose in adults in the U.S. population: National Health And Nutrition Examination Survey 1999–2002.. Diabetes Care.

[2] (2007). Genome-wide association study of 14,000 cases of seven common diseases and 3,000 shared controls.. Nature.

[3] Zeggini E, Scott LJ, Saxena R, Voight BF, Marchini JL (2008). Meta-analysis of genome-wide association data and large-scale replication identifies additional susceptibility loci for type 2 diabetes.. Nat Genet.

[4] Prokopenko I, McCarthy MI, Lindgren CM (2008). Type 2 diabetes: new genes, new understanding.. Trends Genet.

[5] Wild S, Roglic G, Green A, Sicree R, King H (2004). Global prevalence of diabetes: estimates for the year 2000 and projections for 2030.. Diabetes Care.

[6] Freedman BI, Hicks PJ, Bostrom MA, Comeau ME, Divers J (2009). Non-muscle myosin heavy chain 9 gene MYH9 associations in African Americans with clinically diagnosed type 2 diabetes mellitus-associated ESRD.. Nephrol Dial Transplant.

[7] Freedman BI, Hicks PJ, Bostrom MA, Cunningham ME, Liu Y (2009). Polymorphisms in the non-muscle myosin heavy chain 9 gene (MYH9) are strongly associated with end-stage renal disease historically attributed to hypertension in African Americans.. Kidney Int.

[8] Kao WH, Klag MJ, Meoni LA, Reich D, Berthier-Schaad Y (2008). MYH9 is associated with nondiabetic end-stage renal disease in African Americans.. Nat Genet.

[9] Kopp JB, Smith MW, Nelson GW, Johnson RC, Freedman BI (2008). MYH9 is a major-effect risk gene for focal segmental glomerulosclerosis.. Nat Genet.

[10] Altshuler D, Hirschhorn JN, Klannemark M, Lindgren CM, Vohl MC (2000). The common PPARgamma Pro12Ala polymorphism is associated with decreased risk of type 2 diabetes.. Nat Genet.

[11] Bouatia-Naji N, Bonnefond A, Cavalcanti-Proenca C, Sparso T, Holmkvist J (2009). A variant near MTNR1B is associated with increased fasting plasma glucose levels and type 2 diabetes risk.. Nat Genet.

[12] Dupuis J, Langenberg C, Prokopenko I, Saxena R, Soranzo N (2010). New genetic loci implicated in fasting glucose homeostasis and their impact on type 2 diabetes risk.. Nat Genet.

[13] Gloyn AL, Weedon MN, Owen KR, Turner MJ, Knight BA (2003). Large-scale association studies of variants in genes encoding the pancreatic beta-cell KATP channel subunits Kir6.2 (KCNJ11) and SUR1 (ABCC8) confirm that the KCNJ11 E23K variant is associated with type 2 diabetes.. Diabetes.

[14] Grant SF, Thorleifsson G, Reynisdottir I, Benediktsson R, Manolescu A (2006). Variant of transcription factor 7-like 2 (TCF7L2) gene confers risk of type 2 diabetes.. Nat Genet.

[15] Lyssenko V, Nagorny CL, Erdos MR, Wierup N, Jonsson A (2009). Common variant in MTNR1B associated with increased risk of type 2 diabetes and impaired early insulin secretion.. Nat Genet.

[16] Prokopenko I, Langenberg C, Florez JC, Saxena R, Soranzo N (2009). Variants in MTNR1B influence fasting glucose levels.. Nat Genet.

[17] Rung J, Cauchi S, Albrechtsen A, Shen L, Rocheleau G (2009). Genetic variant near IRS1 is associated with type 2 diabetes, insulin resistance and hyperinsulinemia.. Nat Genet.

[18] Sandhu MS, Weedon MN, Fawcett KA, Wasson J, Debenham SL (2007). Common variants in WFS1 confer risk of type 2 diabetes.. Nat Genet.

[19] Saxena R, Voight BF, Lyssenko V, Burtt NP, de Bakker PI (2007). Genome-wide association analysis identifies loci for type 2 diabetes and triglyceride levels.. Science.

[20] Scott LJ, Mohlke KL, Bonnycastle LL, Willer CJ, Li Y (2007). A genome-wide association study of type 2 diabetes in Finns detects multiple susceptibility variants.. Science.

[21] Sladek R, Rocheleau G, Rung J, Dina C, Shen L (2007). A genome-wide association study identifies novel risk loci for type 2 diabetes.. Nature.

[22] Steinthorsdottir V, Thorleifsson G, Reynisdottir I, Benediktsson R, Jonsdottir T (2007). A variant in CDKAL1 influences insulin response and risk of type 2 diabetes.. Nat Genet.

[23] Unoki H, Takahashi A, Kawaguchi T, Hara K, Horikoshi M (2008). SNPs in KCNQ1 are associated with susceptibility to type 2 diabetes in East Asian and European populations.. Nat Genet.

[24] Voight BF, Scott LJ, Steinthorsdottir V, Morris AP, Dina C (2010). Twelve type 2 diabetes susceptibility loci identified through large-scale association analysis.. Nat Genet.

[25] Winckler W, Weedon MN, Graham RR, McCarroll SA, Purcell S (2007). Evaluation of common variants in the six known maturity-onset diabetes of the young (MODY) genes for association with type 2 diabetes.. Diabetes.

[26] Yasuda K, Miyake K, Horikawa Y, Hara K, Osawa H (2008). Variants in KCNQ1 are associated with susceptibility to type 2 diabetes mellitus.. Nat Genet.

[27] Zeggini E, Weedon MN, Lindgren CM, Frayling TM, Elliott KS (2007). Replication of genome-wide association signals in UK samples reveals risk loci for type 2 diabetes.. Science.

[28] Lewis JP, Palmer ND, Hicks PJ, Sale MM, Langefeld CD (2008). Association analysis in african americans of European-derived type 2 diabetes single nucleotide polymorphisms from whole-genome association studies.. Diabetes.

[29] Palmer ND, Goodarzi MO, Langefeld CD, Ziegler J, Norris JM (2008). Quantitative trait analysis of type 2 diabetes susceptibility loci identified from whole genome association studies in the Insulin Resistance Atherosclerosis Family Study.. Diabetes.

[30] Foster R, Hu KQ, Lu Y, Nolan KM, Thissen J (1996). Identification of a novel human Rho protein with unusual properties: GTPase deficiency and in vivo farnesylation.. Mol Cell Biol.

[31] Nobes CD, Lauritzen I, Mattei MG, Paris S, Hall A (1998). A new member of the Rho family, Rnd1, promotes disassembly of actin filament structures and loss of cell adhesion.. J Cell Biol.

[32] Ichikawa K, Yoshinari M, Iwase M, Wakisaka M, Doi Y (1998). Advanced glycosylation end products induced tissue factor expression in human monocyte-like U937 cells and increased tissue factor expression in monocytes from diabetic patients.. Atherosclerosis.

[33] Lim HS, Blann AD, Lip GY (2004). Soluble CD40 ligand, soluble P-selectin, interleukin-6, and tissue factor in diabetes mellitus: relationships to cardiovascular disease and risk factor intervention.. Circulation.

[34] Lim HS, Chong AY, Freestone B, Blann AD, Lip GY (2005). The effect of multi-factorial intervention on plasma von Willebrand factor, soluble E-selectin and tissue factor in diabetes mellitus: implications for atherosclerotic vascular disease.. Diabet Med.

[35] Willer CJ, Sanna S, Jackson AU, Scuteri A, Bonnycastle LL (2008). Newly identified loci that influence lipid concentrations and risk of coronary artery disease.. Nat Genet.

[36] Herring BP, Kriegel AM, Hoggatt AM (2001). Identification of Barx2b, a serum response factor-associated homeodomain protein.. J Biol Chem.

[37] Meech R, Edelman DB, Jones FS, Makarenkova HP (2005). The homeobox transcription factor Barx2 regulates chondrogenesis during limb development.. Development.

[38] Meech R, Makarenkova H, Edelman DB, Jones FS (2003). The homeodomain protein Barx2 promotes myogenic differentiation and is regulated by myogenic regulatory factors.. J Biol Chem.

[39] Florez JC (2007). The new type 2 diabetes gene TCF7L2.. Curr Opin Clin Nutr Metab Care.

[40] Sale MM, Smith SG, Mychaleckyj JC, Keene KL, Langefeld CD (2007). Variants of the transcription factor 7-like 2 (TCF7L2) gene are associated with type 2 diabetes in an African-American population enriched for nephropathy.. Diabetes.

[41] Frazer KA, Ballinger DG, Cox DR, Hinds DA, Stuve LL (2007). A second generation human haplotype map of over 3.1 million SNPs.. Nature.

[42] Huang L, Li Y, Singleton AB, Hardy JA, Abecasis G (2009). Genotype-imputation accuracy across worldwide human populations.. Am J Hum Genet.

[43] McDonough CW, Palmer ND, Hicks PJ, Roh BH, An SS (2010). A genome-wide association study for diabetic nephropathy genes in African Americans.. Kidney Int.

[44] Wagenknecht LE, Mayer EJ, Rewers M, Haffner S, Selby J (1995). The insulin resistance atherosclerosis study (IRAS) objectives, design, and recruitment results.. Ann Epidemiol.

[45] Henkin L, Bergman RN, Bowden DW, Ellsworth DL, Haffner SM (2003). Genetic epidemiology of insulin resistance and visceral adiposity. The IRAS Family Study design and methods.. Ann Epidemiol.

[46] Tang H, Peng J, Wang P, Risch NJ (2005). Estimation of individual admixture: analytical and study design considerations.. Genet Epidemiol.

[47] Keene KL, Mychaleckyj JC, Leak TS, Smith SG, Perlegas PS (2008). Exploration of the utility of ancestry informative markers for genetic association studies of African Americans with type 2 diabetes and end stage renal disease.. Hum Genet.

[48] Harley JB, Alarcon-Riquelme ME, Criswell LA, Jacob CO, Kimberly RP (2008). Genome-wide association scan in women with systemic lupus erythematosus identifies susceptibility variants in ITGAM, PXK, KIAA1542 and other loci.. Nat Genet.

[49] Gabriel SB, Schaffner SF, Nguyen H, Moore JM, Roy J (2002). The structure of haplotype blocks in the human genome.. Science.

[50] Almasy L, Blangero J (1998). Multipoint quantitative-trait linkage analysis in general pedigrees.. Am J Hum Genet.

[51] O'Connell JR, Weeks DE (1998). PedCheck: a program for identification of genotype incompatibilities in linkage analysis.. Am J Hum Genet.

[52] Fisher RA, Immer FR, Tedin O (1932). The Genetical Interpretation of Statistics of the Third Degree in the Study of Quantitative Inheritance.. Genetics.

[53] Whitlock MC (2005). Combining probability from independent tests: the weighted Z-method is superior to Fisher's approach.. J Evol Biol.

[54] Stranger BE, Nica AC, Forrest MS, Dimas A, Bird CP (2007). Population genomics of human gene expression.. Nat Genet.

