# Supplementary figures and images for: A Genome-Wide Association Search for Type 2 Diabetes Genes in African Americans
Source: PLoS One. 2012 Jan 4;7(1):e29202. doi: 10.1371/journal.pone.0029202 (PMC3251563; doi:10.1371/journal.pone.0029202)

**Supplementary Figure 1.** Quartile-Quartile plot of the genome-wide association study results.

**
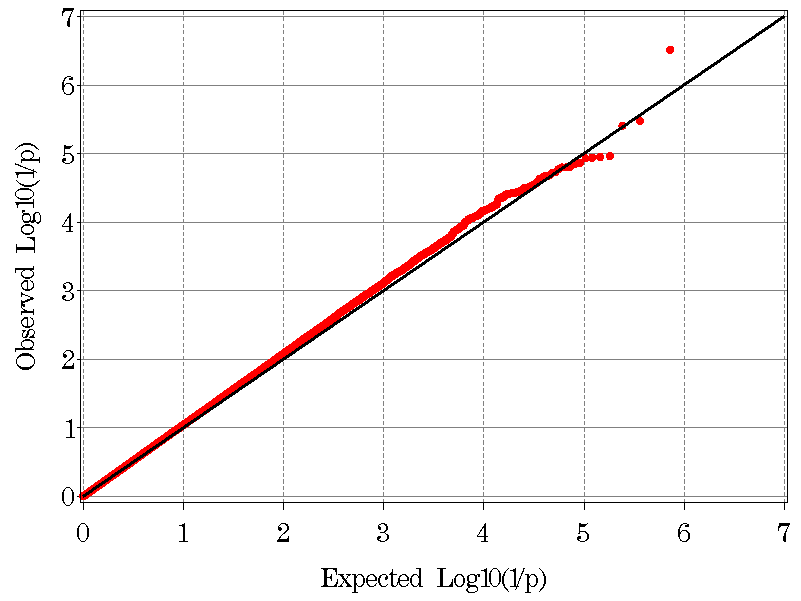
**

Supplement: Figure S1 — Quartile-Quartile plot of the genome-wide association study results. (DOC) [file pone.0029202.s001.doc]
