# Supplementary material for: A Genome-Wide Association Search for Type 2 Diabetes Genes in African Americans
Source: PLoS One. 2012 Jan 4;7(1):e29202. doi: 10.1371/journal.pone.0029202 (PMC3251563; doi:10.1371/journal.pone.0029202)

**Supplementary Figure 2. African-American T2DM candidate regions.** A) rs7542900 region. B) rs4659485 region. C) rs7560163 region. D) rs2722769 region. E) rs7107217 region. –log_10_ additive *P-value* from the GWAS are plotted versus position (NCBI Build 36.1, hg18). The large red diamond indicates the additive *P-value* from the GWAS of the marker(s) displayed. The large blue diamond and corresponding *P-value* indicates the additive *P-values* from the Overall analysis of the marker(s) displayed. r^2^ based on the control samples is color-coded with respect to the most significant SNP: red (0.8-1.0), orange (0.5-0.8), yellow (0.2-0.5) and white (<0.2). Gene annotations were obtained from UCSC Genome Browser (RefSeq Genes, b36). Arrows represent direction of transcription.


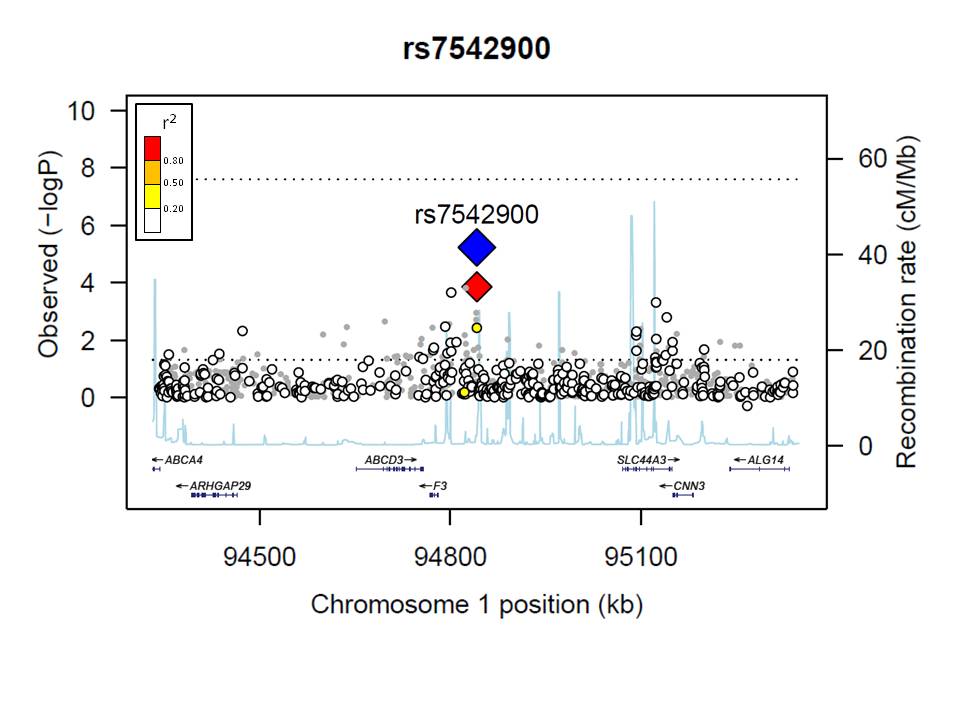


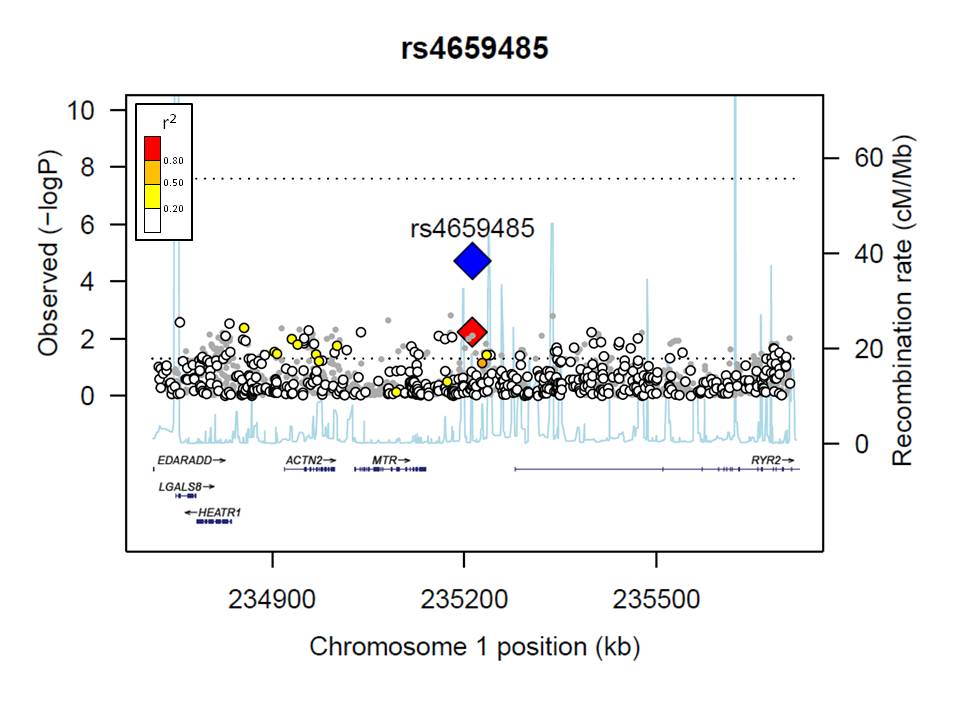


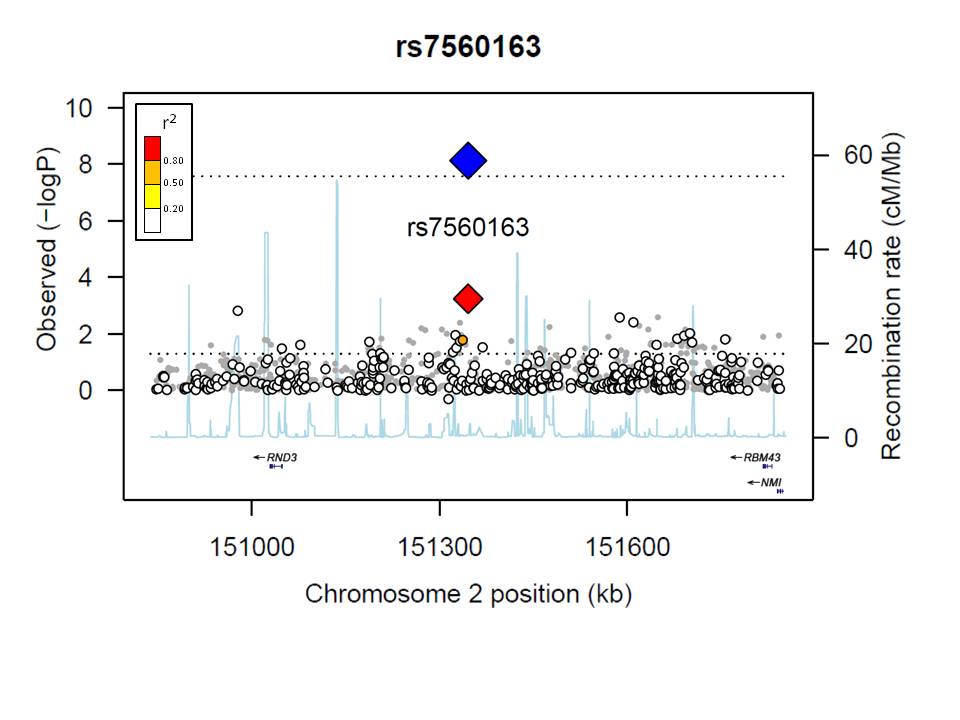


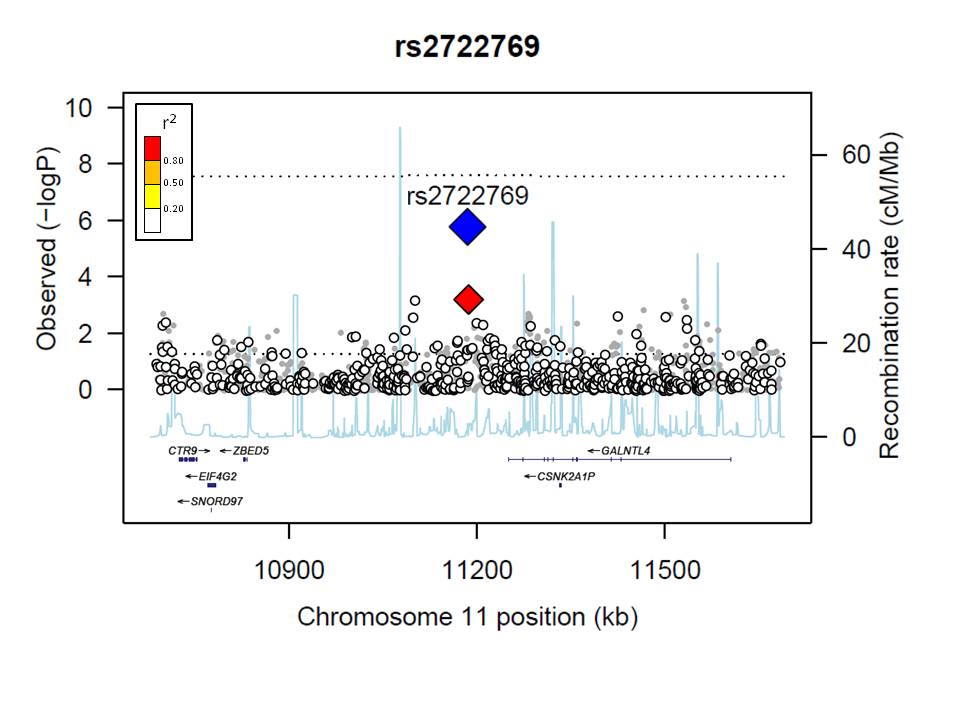


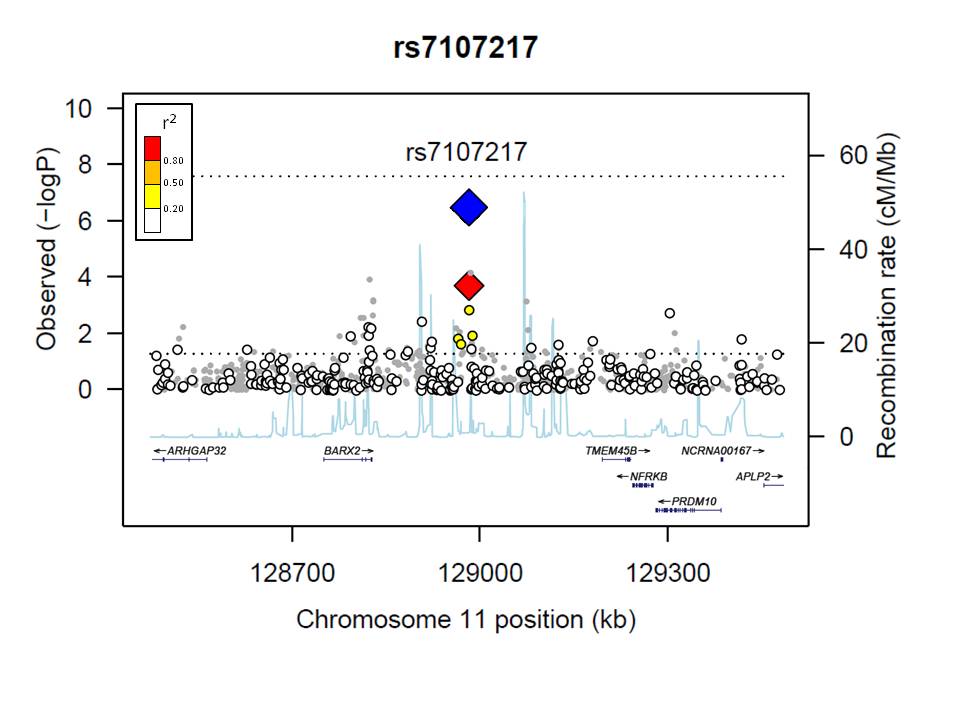

Supplement: Figure S2 — African-American T2DM candidate regions. A) rs7542900 region. B) rs4659485 region. C) rs7560163 region. D) rs2722769 region. E) rs7107217 region. −log10 additive P-value from the GWAS are plotted versus position (NCBI Build 36.1, hg18). The large red diamond indicates the additive P-value from the GWAS of the marker(s) displayed. The large blue diamond and corresponding P-value indicates the additive P-values from the Overall analysis of the marker(s) displayed. r2 based on the control samples is color-coded with respect to the most significant SNP: red (0.8–1.0), orange (0.5–0.8), yellow (0.2–0.5) and white (<0.2). Gene annotations were obtained from UCSC Genome Browser (RefSeq Genes, b36). Arrows represent direction of transcription. (DOC) [file pone.0029202.s002.doc]
