# Supplementary material for: A Genome-Wide Association Search for Type 2 Diabetes Genes in African Americans
Source: PLoS One. 2012 Jan 4;7(1):e29202. doi: 10.1371/journal.pone.0029202 (PMC3251563; doi:10.1371/journal.pone.0029202)
Supplement: Table S1 — GWAS P-values for previously associated T2DM loci. Loci are ordered by chromosome and position (NCBI Build 36.1, hg18) and referenced (Ref) by the initial publication. The African American major/minor alleles are presented on the positive strand with the Caucasian risk allele underlined. For each T2DM Index SNP, results from the African American GWAS (the minor allele frequency (MAF) for the T2DM-ESRD and control populations or combined for imputed SNPs with the corresponding additive P-value and odds ratio (OR) with associated 95% confidence interval (CI)) are presented with respect to the published risk allele (underlined). In addition, association results (additive P-value and odds ratio (OR) with associated 95% confidence interval (CI)) from recent Caucasian large-scale meta-analyses with associated references (Ref) are listed for comparison. For each index SNP, the corresponding LD block was identified using the HapMap phase II CEU data as defined by Gabriel et al. and implemented in Haploview. These intervals were then extracted from the African-American GWAS and the most significant SNP listed. From the GWAS, the minor allele frequency (MAF) for the T2DM-ESRD and control populations are listed with the corresponding additive P-value (nominal and corrected for the effective number of tests at the locus (number of SNPs genotyped in the GWAS and effective number of SNPs determined from the Li and Ji method and implemented in SOLAR)) and odds ratio (OR) with associated 95% confidence interval (CI) with respect to the African-American minor allele. (DOC) [file pone.0029202.s003.doc]

**Supplementary Table 1. GWAS *P-values* for previously associated T2DM loci.** Loci are ordered by chromosome and position (NCBI Build 36.1, hg18) and referenced (Ref) by the initial publication. The African American major/minor alleles are presented on the positive strand with the Caucasian risk allele underlined. For each T2DM Index SNP, results from the African American GWAS (the minor allele frequency (MAF) for the T2DM-ESRD and control populations or combined for imputed SNPs with the corresponding additive *P-value* and odds ratio (OR) with associated 95% confidence interval (CI)) are presented with respect to the published risk allele (underlined). In addition, association results (additive *P-value* and odds ratio (OR) with associated 95% confidence interval (CI)) from recent Caucasian large-scale meta-analyses with associated references (Ref) are listed for comparison. For each index SNP, the corresponding LD block was identified using the HapMap phase II CEU data as defined by Gabriel *et al.* and implemented in Haploview. These intervals were then extracted from the African-American GWAS and the most significant SNP listed. From the GWAS, the minor allele frequency (MAF) for the T2DM-ESRD and control populations are listed with the corresponding additive *P-value* (nominal and corrected for the effective number of tests at the locus (number of SNPs genotyped in the GWAS and effective number of SNPs determined from the Li and Ji method and implemented in SOLAR)) and odds ratio (OR) with associated 95% confidence interval (CI) with respect to the African-American minor allele.

| **Locus** | | | | | | |  | **GWAS** | | | |  | **Caucasian Large-Scale Meta-Analyses** | | |
| --- | --- | --- | --- | --- | --- | --- | --- | --- | --- | --- | --- | --- | --- | --- | --- |
|  |  |  |  |  |  |  |  | **T2DM-ESRD (n=965)** | | | |  |  |  |  |
|  |  |  |  |  |  |  |  | **Control (n=1,029)** | | | |  |  |  |  |
| **T2DM Index SNP** | | **African American SNP** |  |  |  |  |  | **MAF** | | **Additive P-Value** | **OR** |  | **Additive P-Value** | **OR** |  |
| **SNP** | **Ref** |  | **Chr** | **Position** | **Alleles** | **Nearest Gene(s)** |  | **T2DM-ESRD** | **Control** |  | **(95% CI)** |  |  | **(95% CI)** | **Ref** |
| rs10923931 |  |  | 1 | 120319482 | G/T | *NOTCH2* |  | 0.34 | 0.36 | 0.24 | 0.93  (0.81-1.05) |  | 4.1E-08 | 1.13  (1.08-1.17) |  |
|  |  | rs2793823 | 1 | 120239241 | A/G | *ADAM30* |  | 0.39 | 0.36 | 0.025  0.31 (24/12) | 1.16  (1.02-1.33) |  |  |  |  |
| rs340874^b^ |  |  | 1 | 212225879 | T/C |  |  | 0.016 | | 0.26 | 0.90  (0.75-1.08) |  | 7.2E-10 | 1.07  (1.05-1.09) |  |
|  |  | rs340835 | 1 | 212230298 | G/A |  |  | 0.21 | 0.22 | 0.67  0.67 (1/1) | 0.97  (0.83-1.13) |  |  |  |  |
| rs780094 |  |  | 2 | 27594741 | C/T |  |  | 0.15 | 0.17 | 0.21 | 1.11  (0.94-1.31) |  | 1.3E-09 | 1.06  (1.04-1.08) |  |
|  |  | rs780094 | 2 | 27594741 | C/T |  |  | 0.15 | 0.17 | 0.21  0.63 (3/3) | 0.90  (0.76-1.06) |  |  |  |  |
| rs7578597^a^ |  |  | 2 | 43586327 | T/C | *THADA* |  | 0.26 | 0.24 | 0.16 | 1.11  (0.96-1.29) |  | 5.4E-20 | 1.11  (1.08-1.13) |  |
|  |  | rs17334980 | 2 | 43572097 | T/C | *THADA* |  | 0.07 | 0.10 | 0.020  0.24 (22/12) | 0.76  (0.60-0.96) |  |  |  |  |
| rs243021^b^ |  |  | 2 | 60438323 | G/A | *BCL11A* |  | 0.39 | | 0.12 | 0.90  (0.80-1.03) |  | 2.9E-15 | 1.08  (1.06-1.10) |  |
|  |  | rs243019 | 2 | 60439310 | T/C | *BCL11A* |  | 0.39 | 0.42 | 0.13  0.26 (3/2) | 0.91  (0.80-1.03) |  |  |  |  |
| rs2943641^a^ |  |  | 2 | 226801989 | C/T | *IRS1* |  | 0.29 | 0.31 | 0.30 | 0.93  (0.81-1.07) |  | 9.3E-12 | 1.19  (1.13-1.25) |  |
|  |  | rs11675231 | 2 | 226841218 | A/G | *IRS1* |  | 0.09 | 0.07 | 0.056  0.83 (29/15) | 1.25  (0.99-1.57) |  |  |  |  |
| rs1801282 |  |  | 3 | 12368125 | C/G | *PPARG* |  | 0.02 | 0.02 | 0.43 | 0.81  (0.49-1.35) |  | 8.0E-06 | 1.15  (1.08-1.22) | e |
|  |  | rs9817428 | 3 | 12315267 | C/A | *PPARG* |  | 0.35 | 0.39 | 0.0097  0.13 (24/13) | 0.84  (0.74-0.96) |  |  |  |  |
| rs4607103 |  |  | 3 | 64686944 | C/T | *ADAMTS9/MAGI1* |  | 0.30 | 0.28 | 0.22 | 0.92  (0.81-1.05) |  | 1.2E-08 | 1.09  (1.06-1.12) |  |
|  |  | rs9809791 | 3 | 64698672 | G/C | *LOC730057* |  | 0.29 | 0.32 | 0.030  0.33 (21/11) | 0.86  (0.75-0.99) |  |  |  |  |
| rs11708067 |  |  | 3 | 124548468 | A/G |  |  | 0.14 | 0.14 | 0.91 | 1.01  (0.85-1.21) |  | 9.9E-21 | 1.12  (1.09-1.15) |  |
|  |  | rs6807089 | 3 | 124546779 | T/C |  |  | 0.23 | 0.23 | 0.75  2.99 (5/4) | 1.02  (0.88-1.19) |  |  |  |  |
| rs4402960 |  |  | 3 | 186994381 | T/G | *IGF2BP2* |  | 0.47 | 0.47 | 0.66 | 0.97  (0.86-1.10) |  | 7.5E-08 | 1.17  (1.10-1.25) | e |
|  |  | rs764128 | 3 | 187030527 | G/A | *TRA2B/IGF2BP2* |  | 0.15 | 0.12 | 0.017  0.19 (21/11) | 1.25  (1.04-1.50) |  |  |  |  |
| rs10010131^a^ |  |  | 4 | 6343816 | G/A | *WFS1* |  | 0.33 | 0.35 | 0.21 | 0.92  (0.80-1.05) |  | 4.6E-07 | 1.11  (1.07-1.16) | e |
|  |  | rs1801212 | 4 | 6353420 | A/G | *WFS1* |  | 0.03 | 0.05 | 0.0041  0.058 (22/14) | 0.59  (0.41-0.85) |  |  |  |  |
| rs4457053^cd^ |  |  | 5 | 76460705 |  | *ZED3* |  | - | - | - | - |  | 2.8E-12 | 1.08  (1.06-1.11) |  |
|  |  |  |  |  |  |  |  |  |  |  |  |  |  |  |  |
| rs10946398 |  |  | 6 | 20769013 | C/A | *CDKAL1* |  | 0.37 | 0.40 | 0.11 | 1.11  (0.98-1.27) |  | 4.1E-11 | 1.12  (1.08-1.16) |  |
|  |  | rs4712527 | 6 | 20771314 | A/G | *CDKAL1* |  | 0.08 | 0.10 | 0.035  0.21 (11/6) | 0.79  (0.63-0.98) |  |  |  |  |
| rs864745 |  |  | 7 | 28147081 | T/C | *JAZF1* |  | 0.22 | 0.24 | 0.090 | 1.14  (0.98-1.27) |  | 5.0E-14 | 1.10  (1.07-1.13) |  |
|  |  | rs552707 | 7 | 28171828 | C/T | *JAZF1* |  | 0.12 | 0.14 | 0.048  0.33 (11/7) | 0.83  (0.69-1.00) |  |  |  |  |
| rs2191349^b^ |  |  | 7 | 15030834 | T/G |  |  | 0.39 | | 0.71 | 0.98  (0.85-1.11) |  | 1.1E-08 | 1.06  (1.04-1.08) |  |
|  |  | rs6947830 | 7 | 15064984 | A/G |  |  | 0.38 | 0.39 | 0.91  2.74 (5/3) | 0.99  (0.87-1.13) |  |  |  |  |
| rs4607517^b^ |  |  | 7 | 44202193 | G/A |  |  | 0.10 | | 0.80 | 0.97  (0.77-1.22) |  | 5.0E-08 | 1.07  (1.05-1.10) |  |
|  |  | rs741038 | 7 | 44190004 | A/G |  |  | 0.25 | 0.24 | 0.43  1.70 (5/4) | 1.06  (0.92-1.22) |  |  |  |  |
| rs972283^b^ |  |  | 7 | 130117394 | G/A | *KLF14* |  | 0.14 | | 0.67 | 0.96  (0.78-1.17) |  | 2.2E-10 | 1.07  (1.05-1.10) |  |
|  |  | rs738134 | 7 | 130118115 | C/G | *MIR29A/KLF14* |  | 0.26 | 0.26 | 0.89  0.89 (1/1) | 0.99  (0.86-1.14) |  |  |  |  |
| rs896854^b^ |  |  | 8 | 96029687 | T/C | *TP53INP1* |  | 0.29 | | 0.45 | 0.95  (0.82-1.09) |  | 9.9E-10 | 1.06  (1.04-1.09) |  |
|  |  | rs1713669 | 8 | 96027813 | C/G | *TP53INP1* |  | 0.22 | 0.21 | 0.51  1.02 (3/2) | 1.05  (0.90-1.23) |  |  |  |  |
| rs13266634^a^ |  |  | 8 | 118253964 | C/T | *SLC30A8* |  | 0.07 | 0.08 | 0.36 | 0.89  (0.70-1.14) |  | 1.5E-08 | 1.15  (1.10-1.21) |  |
|  |  | rs2466296 | 8 | 118253308 | A/G | *SLC30A8* |  | 0.33 | 0.35 | 0.47  0.47 (1/1) | 0.95  (0.83-1.09) |  |  |  |  |
| rs10811661 |  |  | 9 | 22124094 | T/C | *CDKN2B/DMRTA1* |  | 0.07 | 0.07 | 0.67 | 0.95  (0.73-1.22) |  | 1.5E-10 | 1.19  (1.13-1.26) | e |
|  |  | rs2383208 | 9 | 22122076 | A/G | *DMRTA1/CDKN2BAS* |  | 0.20 | 0.19 | 0.24  1.24 (7/6) | 1.10  (0.94-1.28) |  |  |  |  |
| rs13292136^b^ |  |  | 9 | 81141948 | C/T | *CHCHD9* |  | 0.09 | | 0.97 | 1.01  (0.81-1.25) |  | 2.8E-08 | 1.11  (1.07-1.15) |  |
|  |  | rs10780301 | 9 | 81120294 | A/G | *TLE4* |  | 0.16 | 0.19 | 0.030  0.18 (11/6) | 0.83  (0.71-0.98) |  |  |  |  |
| rs12779790^a^ |  |  | 10 | 12368016 | A/G | *CDC123/CAMK1D* |  | 0.15 | 0.14 | 0.63 | 1.05  (0.87-1.25) |  | 1.2E-10 | 1.11  (1.07-1.14) |  |
|  |  | rs7069060 | 10 | 12363470 | A/G | *CAMK1D/CDC123* |  | 0.27 | 0.25 | 0.25  0.51 (2/2) | 1.09  (0.94-1.25) |  |  |  |  |
| rs1111875 |  |  | 10 | 94452862 | C/T | *HHEX/EXOC6* |  | 0.22 | 0.22 | 0.83 | 1.02  (0.88-1.18) |  | 9.1E-15 | 1.17  (1.12-1.22) | e |
|  |  | rs5015480 | 10 | 94455539 | C/T | *EXOC6/HHEX* |  | 0.39 | 0.37 | 0.25  1.27 (6/5) | 1.08  (0.95-1.23) |  |  |  |  |
| rs7903146^a^ |  |  | 10 | 114748339 | C/T | *TCF7L2* |  | 0.34 | 0.28 | 4.9E-05 | 1.33  (1.16-1.53) |  | 2.2E-51 | 1.40  (1.34-1.46) |  |
|  |  | rs4506565 | 10 | 114746031 | A/T | *TCF7L2* |  | 0.50 | 0.45 | 0.0015  0.015 (18/10) | 1.23  (1.08-1.39) |  |  |  |  |
| rs231362^bd^ |  |  | 11 | 2648047 | G/A | *KCNQ1* |  | 0.21 | | 0.68 | 0.96  (0.80-1.15) |  | 2.8E-13 | 1.08  (1.06-1.10) |  |
|  |  |  |  |  |  |  |  |  |  |  |  |  |  |  |  |
| rs2237892^a^ |  |  | 11 | 2796327 | C/T | *KCNQ1* |  | 0.09 | 0.10 | 0.96 | 0.99  (0.80-1.23) |  | 0.0027 | 1.14  (1.05-1.24) | e |
|  |  | rs163183 | 11 | 2801017 | C/T | *KCNQ1* |  | 0.37 | 0.36 | 0.37  1.47 (4/4) | 1.06  (0.93-1.21) |  |  |  |  |
| rs5215 |  |  | 11 | 17365206 | T/C | *KCNJ11* |  | 0.08 | 0.09 | 0.45 | 0.92  (0.73-1.15) |  | 1.6E-05 | 1.09  (1.05-1.14) | e |
|  |  | rs7110094 | 11 | 17358095 | A/G | *KCNJ11/DKFZp686O24166* |  | 0.49 | 0.48 | 0.20  1.37 (11/7) | 1.09  (0.96-1.23) |  |  |  |  |
| rs1552224^b^ |  |  | 11 | 72110746 | A/C | *CENTD2* |  | 0.03 | | 0.32 | 1.21  (0.83-1.77) |  | 1.4E-22 | 1.14  (1.11-1.17) |  |
|  |  | rs10898869 | 11 | 72109601 | C/T | *CENTD2* |  | 0.16 | 0.18 | 0.036  0.072 (4/2) | 0.84  (0.71-0.99) |  |  |  |  |
| rs10830963 |  |  | 11 | 92348358 | C/G | *MTNR1B* |  | 0.07 | 0.07 | 0.51 | 1.09  (0.84-1.40) |  | 8.0E-13 | 1.09  (1.06-1.12) |  |
|  |  | rs12272268 | 11 | 92348447 | C/G | *MTNR1B* |  | 0.26 | 0.27 | 0.18  1.06 (9/6) | 0.91  (0.79-1.05) |  |  |  |  |
| rs1531343 |  |  | 12 | 64461161 | G/C | *RPSAP52* |  | 0.39 | 0.38 | 0.78 | 1.02  (0.90-1.16) |  | 3.6E-09 | 1.10  (1.07-1.14) |  |
|  |  | rs2260671 | 12 | 64461176 | G/A | *HMGA2/MSRB3* |  | 0.05 | 0.06 | 0.20  2.03 (17/10) | 0.84  (0.65-1.10) |  |  |  |  |
| rs7961581 |  |  | 12 | 69949369 | T/C | *TSPAN8* |  | 0.19 | 0.20 | 0.92 | 1.01  (0.86-1.18) |  | 1.1E-09 | 1.09  (1.06-1.12) |  |
|  |  | rs41391144 | 12 | 69946900 | C/T | *LGR5/TSPAN8* |  | 0.01 | 0.02 | 0.030  0.064 (2/2) | 0.50  (0.26-0.94) |  |  |  |  |
| rs7957197^bd^ |  |  | 12 | 119945069 | T/A | *HNF1A* |  | 0.14 | | 0.73 | 0.97  (0.80-1.17) |  | 2.4E-08 | 1.07  (1.05-1.10) |  |
|  |  |  |  |  |  |  |  |  |  |  |  |  |  |  |  |
| rs11634397 |  |  | 15 | 78219277 | A/G | *ZFAND6/FAH* |  | 0.42 | 0.46 | 0.016 | 0.86  (0.75-0.97) |  | 2.4E-09 | 1.06  (1.04-1.08) |  |
|  |  | rs11634397 | 15 | 78219277 | A/G | *ZFAND6/FAH* |  | 0.42 | 0.46 | 0.016  0.063 (6/4) | 0.86  (0.75-0.97) |  |  |  |  |
| rs8042680 |  |  | 15 | 89322341 | A/C | *PRC1* |  | 0.14 | 0.14 | 0.33 | 0.91  (0.76-1.09) |  | 2.4E-10 | 1.07  (1.05-1.09) |  |
|  |  | rs2290203 | 15 | 89313071 | G/A | *PRC1* |  | 0.43 | 0.40 | 0.16  0.96 (7/6) | 1.10  (0.96-1.25) |  |  |  |  |
| rs8050136 |  |  | 16 | 52373776 | C/A | *FTO* |  | 0.44 | 0.46 | 0.42 | 0.95  (0.84-1.08) |  | 6.9E-06 | 1.15  (1.09-1.22) | e |
|  |  | rs17817288 | 16 | 52365265 | A/G | *FTO* |  | 0.43 | 0.40 | 0.061  0.74 (22/12) | 1.13  (0.99-1.28) |  |  |  |  |
| rs4430796^a^ |  |  | 17 | 33172153 | G/A | *TCF2* |  | 0.31 | 0.33 | 0.33 | 0.93  (0.81-1.07) |  | 1.5E-06 | 1.14  (1.08-1.20) | e |
|  |  | rs11651755 | 17 | 33173953 | C/T | *HNF1B* |  | 0.32 | 0.33 | 0.42  0.83 (3/2) | 0.95  (0.82-1.08) |  |  |  |  |
| rs5945326^cd^ |  |  | X | 152553116 |  | *DUSP9* |  | - | - | - | - |  | 3.0E-10 | 1.27  (1.18-1.37) |  |
|  |  |  |  |  |  |  |  |  |  |  |  |  |  |  |  |

^a^SNPs not available on the Affymetrix 6.0 array. Genotypes were obtained from de novo genotyping on the Sequenom Mass Array.

^b^SNPs not available on the Affymetrix 6.0 array. Genotypes were obtained from imputation.

^c^SNPs not available on the Affymetrix 6.0 array.

^d^Index SNP containing CEU LD block contained no SNPs genotyped on the Affymetrix 6.0 array.

^e^Results reported from Stage 1 of the referenced study.

**References**

1. Zeggini E, Scott LJ, Saxena R, Voight BF, Marchini JL, Hu T, de Bakker PI, Abecasis GR, Almgren P, Andersen G, Ardlie K, Bostrom KB, Bergman RN, Bonnycastle LL, Borch-Johnsen K, Burtt NP, Chen H, Chines PS, Daly MJ, Deodhar P, Ding CJ, Doney AS, Duren WL, Elliott KS, Erdos MR, Frayling TM, Freathy RM, Gianniny L, Grallert H, Grarup N, Groves CJ, Guiducci C, Hansen T, Herder C, Hitman GA, Hughes TE, Isomaa B, Jackson AU, Jorgensen T, Kong A, Kubalanza K, Kuruvilla FG, Kuusisto J, Langenberg C, Lango H, Lauritzen T, Li Y, Lindgren CM, Lyssenko V, Marvelle AF, Meisinger C, Midthjell K, Mohlke KL, Morken MA, Morris AD, Narisu N, Nilsson P, Owen KR, Palmer CN, Payne F, Perry JR, Pettersen E, Platou C, Prokopenko I, Qi L, Qin L, Rayner NW, Rees M, Roix JJ, Sandbaek A, Shields B, Sjogren M, Steinthorsdottir V, Stringham HM, Swift AJ, Thorleifsson G, Thorsteinsdottir U, Timpson NJ, Tuomi T, Tuomilehto J, Walker M, Watanabe RM, Weedon MN, Willer CJ, Illig T, Hveem K, Hu FB, Laakso M, Stefansson K, Pedersen O, Wareham NJ, Barroso I, Hattersley AT, Collins FS, Groop L, McCarthy MI, Boehnke M, Altshuler D: Meta-analysis of genome-wide association data and large-scale replication identifies additional susceptibility loci for type 2 diabetes. *Nat Genet* 40:638-645, 2008

2. Dupuis J, Langenberg C, Prokopenko I, Saxena R, Soranzo N, Jackson AU, Wheeler E, Glazer NL, Bouatia-Naji N, Gloyn AL, Lindgren CM, Magi R, Morris AP, Randall J, Johnson T, Elliott P, Rybin D, Thorleifsson G, Steinthorsdottir V, Henneman P, Grallert H, Dehghan A, Hottenga JJ, Franklin CS, Navarro P, Song K, Goel A, Perry JR, Egan JM, Lajunen T, Grarup N, Sparso T, Doney A, Voight BF, Stringham HM, Li M, Kanoni S, Shrader P, Cavalcanti-Proenca C, Kumari M, Qi L, Timpson NJ, Gieger C, Zabena C, Rocheleau G, Ingelsson E, An P, O'Connell J, Luan J, Elliott A, McCarroll SA, Payne F, Roccasecca RM, Pattou F, Sethupathy P, Ardlie K, Ariyurek Y, Balkau B, Barter P, Beilby JP, Ben-Shlomo Y, Benediktsson R, Bennett AJ, Bergmann S, Bochud M, Boerwinkle E, Bonnefond A, Bonnycastle LL, Borch-Johnsen K, Bottcher Y, Brunner E, Bumpstead SJ, Charpentier G, Chen YD, Chines P, Clarke R, Coin LJ, Cooper MN, Cornelis M, Crawford G, Crisponi L, Day IN, de Geus EJ, Delplanque J, Dina C, Erdos MR, Fedson AC, Fischer-Rosinsky A, Forouhi NG, Fox CS, Frants R, Franzosi MG, Galan P, Goodarzi MO, Graessler J, Groves CJ, Grundy S, Gwilliam R, Gyllensten U, Hadjadj S, Hallmans G, Hammond N, Han X, Hartikainen AL, Hassanali N, Hayward C, Heath SC, Hercberg S, Herder C, Hicks AA, Hillman DR, Hingorani AD, Hofman A, Hui J, Hung J, Isomaa B, Johnson PR, Jorgensen T, Jula A, Kaakinen M, Kaprio J, Kesaniemi YA, Kivimaki M, Knight B, Koskinen S, Kovacs P, Kyvik KO, Lathrop GM, Lawlor DA, Le Bacquer O, Lecoeur C, Li Y, Lyssenko V, Mahley R, Mangino M, Manning AK, Martinez-Larrad MT, McAteer JB, McCulloch LJ, McPherson R, Meisinger C, Melzer D, Meyre D, Mitchell BD, Morken MA, Mukherjee S, Naitza S, Narisu N, Neville MJ, Oostra BA, Orru M, Pakyz R, Palmer CN, Paolisso G, Pattaro C, Pearson D, Peden JF, Pedersen NL, Perola M, Pfeiffer AF, Pichler I, Polasek O, Posthuma D, Potter SC, Pouta A, Province MA, Psaty BM, Rathmann W, Rayner NW, Rice K, Ripatti S, Rivadeneira F, Roden M, Rolandsson O, Sandbaek A, Sandhu M, Sanna S, Sayer AA, Scheet P, Scott LJ, Seedorf U, Sharp SJ, Shields B, Sigurethsson G, Sijbrands EJ, Silveira A, Simpson L, Singleton A, Smith NL, Sovio U, Swift A, Syddall H, Syvanen AC, Tanaka T, Thorand B, Tichet J, Tonjes A, Tuomi T, Uitterlinden AG, van Dijk KW, van Hoek M, Varma D, Visvikis-Siest S, Vitart V, Vogelzangs N, Waeber G, Wagner PJ, Walley A, Walters GB, Ward KL, Watkins H, Weedon MN, Wild SH, Willemsen G, Witteman JC, Yarnell JW, Zeggini E, Zelenika D, Zethelius B, Zhai G, Zhao JH, Zillikens MC, Borecki IB, Loos RJ, Meneton P, Magnusson PK, Nathan DM, Williams GH, Hattersley AT, Silander K, Salomaa V, Smith GD, Bornstein SR, Schwarz P, Spranger J, Karpe F, Shuldiner AR, Cooper C, Dedoussis GV, Serrano-Rios M, Morris AD, Lind L, Palmer LJ, Hu FB, Franks PW, Ebrahim S, Marmot M, Kao WH, Pankow JS, Sampson MJ, Kuusisto J, Laakso M, Hansen T, Pedersen O, Pramstaller PP, Wichmann HE, Illig T, Rudan I, Wright AF, Stumvoll M, Campbell H, Wilson JF, Bergman RN, Buchanan TA, Collins FS, Mohlke KL, Tuomilehto J, Valle TT, Altshuler D, Rotter JI, Siscovick DS, Penninx BW, Boomsma DI, Deloukas P, Spector TD, Frayling TM, Ferrucci L, Kong A, Thorsteinsdottir U, Stefansson K, van Duijn CM, Aulchenko YS, Cao A, Scuteri A, Schlessinger D, Uda M, Ruokonen A, Jarvelin MR, Waterworth DM, Vollenweider P, Peltonen L, Mooser V, Abecasis GR, Wareham NJ, Sladek R, Froguel P, Watanabe RM, Meigs JB, Groop L, Boehnke M, McCarthy MI, Florez JC, Barroso I: New genetic loci implicated in fasting glucose homeostasis and their impact on type 2 diabetes risk. *Nat Genet* 42:105-116, 2010

3. Voight BF, Scott LJ, Steinthorsdottir V, Morris AP, Dina C, Welch RP, Zeggini E, Huth C, Aulchenko YS, Thorleifsson G, McCulloch LJ, Ferreira T, Grallert H, Amin N, Wu G, Willer CJ, Raychaudhuri S, McCarroll SA, Langenberg C, Hofmann OM, Dupuis J, Qi L, Segre AV, van Hoek M, Navarro P, Ardlie K, Balkau B, Benediktsson R, Bennett AJ, Blagieva R, Boerwinkle E, Bonnycastle LL, Bengtsson Bostrom K, Bravenboer B, Bumpstead S, Burtt NP, Charpentier G, Chines PS, Cornelis M, Couper DJ, Crawford G, Doney AS, Elliott KS, Elliott AL, Erdos MR, Fox CS, Franklin CS, Ganser M, Gieger C, Grarup N, Green T, Griffin S, Groves CJ, Guiducci C, Hadjadj S, Hassanali N, Herder C, Isomaa B, Jackson AU, Johnson PR, Jorgensen T, Kao WH, Klopp N, Kong A, Kraft P, Kuusisto J, Lauritzen T, Li M, Lieverse A, Lindgren CM, Lyssenko V, Marre M, Meitinger T, Midthjell K, Morken MA, Narisu N, Nilsson P, Owen KR, Payne F, Perry JR, Petersen AK, Platou C, Proenca C, Prokopenko I, Rathmann W, Rayner NW, Robertson NR, Rocheleau G, Roden M, Sampson MJ, Saxena R, Shields BM, Shrader P, Sigurdsson G, Sparso T, Strassburger K, Stringham HM, Sun Q, Swift AJ, Thorand B, Tichet J, Tuomi T, van Dam RM, van Haeften TW, van Herpt T, van Vliet-Ostaptchouk JV, Walters GB, Weedon MN, Wijmenga C, Witteman J, Bergman RN, Cauchi S, Collins FS, Gloyn AL, Gyllensten U, Hansen T, Hide WA, Hitman GA, Hofman A, Hunter DJ, Hveem K, Laakso M, Mohlke KL, Morris AD, Palmer CN, Pramstaller PP, Rudan I, Sijbrands E, Stein LD, Tuomilehto J, Uitterlinden A, Walker M, Wareham NJ, Watanabe RM, Abecasis GR, Boehm BO, Campbell H, Daly MJ, Hattersley AT, Hu FB, Meigs JB, Pankow JS, Pedersen O, Wichmann HE, Barroso I, Florez JC, Frayling TM, Groop L, Sladek R, Thorsteinsdottir U, Wilson JF, Illig T, Froguel P, van Duijn CM, Stefansson K, Altshuler D, Boehnke M, McCarthy MI: Twelve type 2 diabetes susceptibility loci identified through large-scale association analysis. *Nat Genet* 42:579-589, 2010

4. Rung J, Cauchi S, Albrechtsen A, Shen L, Rocheleau G, Cavalcanti-Proenca C, Bacot F, Balkau B, Belisle A, Borch-Johnsen K, Charpentier G, Dina C, Durand E, Elliott P, Hadjadj S, Jarvelin MR, Laitinen J, Lauritzen T, Marre M, Mazur A, Meyre D, Montpetit A, Pisinger C, Posner B, Poulsen P, Pouta A, Prentki M, Ribel-Madsen R, Ruokonen A, Sandbaek A, Serre D, Tichet J, Vaxillaire M, Wojtaszewski JF, Vaag A, Hansen T, Polychronakos C, Pedersen O, Froguel P, Sladek R: Genetic variant near IRS1 is associated with type 2 diabetes, insulin resistance and hyperinsulinemia. *Nat Genet* 41:1110-1115, 2009

5. Altshuler D, Hirschhorn JN, Klannemark M, Lindgren CM, Vohl MC, Nemesh J, Lane CR, Schaffner SF, Bolk S, Brewer C, Tuomi T, Gaudet D, Hudson TJ, Daly M, Groop L, Lander ES: The common PPARgamma Pro12Ala polymorphism is associated with decreased risk of type 2 diabetes. *Nat Genet* 26:76-80, 2000

6. Saxena R, Voight BF, Lyssenko V, Burtt NP, de Bakker PI, Chen H, Roix JJ, Kathiresan S, Hirschhorn JN, Daly MJ, Hughes TE, Groop L, Altshuler D, Almgren P, Florez JC, Meyer J, Ardlie K, Bengtsson Bostrom K, Isomaa B, Lettre G, Lindblad U, Lyon HN, Melander O, Newton-Cheh C, Nilsson P, Orho-Melander M, Rastam L, Speliotes EK, Taskinen MR, Tuomi T, Guiducci C, Berglund A, Carlson J, Gianniny L, Hackett R, Hall L, Holmkvist J, Laurila E, Sjogren M, Sterner M, Surti A, Svensson M, Tewhey R, Blumenstiel B, Parkin M, Defelice M, Barry R, Brodeur W, Camarata J, Chia N, Fava M, Gibbons J, Handsaker B, Healy C, Nguyen K, Gates C, Sougnez C, Gage D, Nizzari M, Gabriel SB, Chirn GW, Ma Q, Parikh H, Richardson D, Ricke D, Purcell S: Genome-wide association analysis identifies loci for type 2 diabetes and triglyceride levels. *Science* 316:1331-1336, 2007

7. Scott LJ, Mohlke KL, Bonnycastle LL, Willer CJ, Li Y, Duren WL, Erdos MR, Stringham HM, Chines PS, Jackson AU, Prokunina-Olsson L, Ding CJ, Swift AJ, Narisu N, Hu T, Pruim R, Xiao R, Li XY, Conneely KN, Riebow NL, Sprau AG, Tong M, White PP, Hetrick KN, Barnhart MW, Bark CW, Goldstein JL, Watkins L, Xiang F, Saramies J, Buchanan TA, Watanabe RM, Valle TT, Kinnunen L, Abecasis GR, Pugh EW, Doheny KF, Bergman RN, Tuomilehto J, Collins FS, Boehnke M: A genome-wide association study of type 2 diabetes in Finns detects multiple susceptibility variants. *Science* 316:1341-1345, 2007

8. Zeggini E, Weedon MN, Lindgren CM, Frayling TM, Elliott KS, Lango H, Timpson NJ, Perry JR, Rayner NW, Freathy RM, Barrett JC, Shields B, Morris AP, Ellard S, Groves CJ, Harries LW, Marchini JL, Owen KR, Knight B, Cardon LR, Walker M, Hitman GA, Morris AD, Doney AS, McCarthy MI, Hattersley AT: Replication of genome-wide association signals in UK samples reveals risk loci for type 2 diabetes. *Science* 316:1336-1341, 2007

9. Sandhu MS, Weedon MN, Fawcett KA, Wasson J, Debenham SL, Daly A, Lango H, Frayling TM, Neumann RJ, Sherva R, Blech I, Pharoah PD, Palmer CN, Kimber C, Tavendale R, Morris AD, McCarthy MI, Walker M, Hitman G, Glaser B, Permutt MA, Hattersley AT, Wareham NJ, Barroso I: Common variants in WFS1 confer risk of type 2 diabetes. *Nat Genet* 39:951-953, 2007

10. Steinthorsdottir V, Thorleifsson G, Reynisdottir I, Benediktsson R, Jonsdottir T, Walters GB, Styrkarsdottir U, Gretarsdottir S, Emilsson V, Ghosh S, Baker A, Snorradottir S, Bjarnason H, Ng MC, Hansen T, Bagger Y, Wilensky RL, Reilly MP, Adeyemo A, Chen Y, Zhou J, Gudnason V, Chen G, Huang H, Lashley K, Doumatey A, So WY, Ma RC, Andersen G, Borch-Johnsen K, Jorgensen T, van Vliet-Ostaptchouk JV, Hofker MH, Wijmenga C, Christiansen C, Rader DJ, Rotimi C, Gurney M, Chan JC, Pedersen O, Sigurdsson G, Gulcher JR, Thorsteinsdottir U, Kong A, Stefansson K: A variant in CDKAL1 influences insulin response and risk of type 2 diabetes. *Nat Genet* 39:770-775, 2007

11. Sladek R, Rocheleau G, Rung J, Dina C, Shen L, Serre D, Boutin P, Vincent D, Belisle A, Hadjadj S, Balkau B, Heude B, Charpentier G, Hudson TJ, Montpetit A, Pshezhetsky AV, Prentki M, Posner BI, Balding DJ, Meyre D, Polychronakos C, Froguel P: A genome-wide association study identifies novel risk loci for type 2 diabetes. *Nature* 445:881-885, 2007

12. Grant SF, Thorleifsson G, Reynisdottir I, Benediktsson R, Manolescu A, Sainz J, Helgason A, Stefansson H, Emilsson V, Helgadottir A, Styrkarsdottir U, Magnusson KP, Walters GB, Palsdottir E, Jonsdottir T, Gudmundsdottir T, Gylfason A, Saemundsdottir J, Wilensky RL, Reilly MP, Rader DJ, Bagger Y, Christiansen C, Gudnason V, Sigurdsson G, Thorsteinsdottir U, Gulcher JR, Kong A, Stefansson K: Variant of transcription factor 7-like 2 (TCF7L2) gene confers risk of type 2 diabetes. *Nat Genet* 38:320-323, 2006

13. Unoki H, Takahashi A, Kawaguchi T, Hara K, Horikoshi M, Andersen G, Ng DP, Holmkvist J, Borch-Johnsen K, Jorgensen T, Sandbaek A, Lauritzen T, Hansen T, Nurbaya S, Tsunoda T, Kubo M, Babazono T, Hirose H, Hayashi M, Iwamoto Y, Kashiwagi A, Kaku K, Kawamori R, Tai ES, Pedersen O, Kamatani N, Kadowaki T, Kikkawa R, Nakamura Y, Maeda S: SNPs in KCNQ1 are associated with susceptibility to type 2 diabetes in East Asian and European populations. *Nat Genet* 40:1098-1102, 2008

14. Yasuda K, Miyake K, Horikawa Y, Hara K, Osawa H, Furuta H, Hirota Y, Mori H, Jonsson A, Sato Y, Yamagata K, Hinokio Y, Wang HY, Tanahashi T, Nakamura N, Oka Y, Iwasaki N, Iwamoto Y, Yamada Y, Seino Y, Maegawa H, Kashiwagi A, Takeda J, Maeda E, Shin HD, Cho YM, Park KS, Lee HK, Ng MC, Ma RC, So WY, Chan JC, Lyssenko V, Tuomi T, Nilsson P, Groop L, Kamatani N, Sekine A, Nakamura Y, Yamamoto K, Yoshida T, Tokunaga K, Itakura M, Makino H, Nanjo K, Kadowaki T, Kasuga M: Variants in KCNQ1 are associated with susceptibility to type 2 diabetes mellitus. *Nat Genet* 40:1092-1097, 2008

15. Gloyn AL, Weedon MN, Owen KR, Turner MJ, Knight BA, Hitman G, Walker M, Levy JC, Sampson M, Halford S, McCarthy MI, Hattersley AT, Frayling TM: Large-scale association studies of variants in genes encoding the pancreatic beta-cell KATP channel subunits Kir6.2 (KCNJ11) and SUR1 (ABCC8) confirm that the KCNJ11 E23K variant is associated with type 2 diabetes. *Diabetes* 52:568-572, 2003

16. Bouatia-Naji N, Bonnefond A, Cavalcanti-Proenca C, Sparso T, Holmkvist J, Marchand M, Delplanque J, Lobbens S, Rocheleau G, Durand E, De Graeve F, Chevre JC, Borch-Johnsen K, Hartikainen AL, Ruokonen A, Tichet J, Marre M, Weill J, Heude B, Tauber M, Lemaire K, Schuit F, Elliott P, Jorgensen T, Charpentier G, Hadjadj S, Cauchi S, Vaxillaire M, Sladek R, Visvikis-Siest S, Balkau B, Levy-Marchal C, Pattou F, Meyre D, Blakemore AI, Jarvelin MR, Walley AJ, Hansen T, Dina C, Pedersen O, Froguel P: A variant near MTNR1B is associated with increased fasting plasma glucose levels and type 2 diabetes risk. *Nat Genet* 41:89-94, 2009

17. Lyssenko V, Nagorny CL, Erdos MR, Wierup N, Jonsson A, Spegel P, Bugliani M, Saxena R, Fex M, Pulizzi N, Isomaa B, Tuomi T, Nilsson P, Kuusisto J, Tuomilehto J, Boehnke M, Altshuler D, Sundler F, Eriksson JG, Jackson AU, Laakso M, Marchetti P, Watanabe RM, Mulder H, Groop L: Common variant in MTNR1B associated with increased risk of type 2 diabetes and impaired early insulin secretion. *Nat Genet* 41:82-88, 2009

18. Prokopenko I, Langenberg C, Florez JC, Saxena R, Soranzo N, Thorleifsson G, Loos RJ, Manning AK, Jackson AU, Aulchenko Y, Potter SC, Erdos MR, Sanna S, Hottenga JJ, Wheeler E, Kaakinen M, Lyssenko V, Chen WM, Ahmadi K, Beckmann JS, Bergman RN, Bochud M, Bonnycastle LL, Buchanan TA, Cao A, Cervino A, Coin L, Collins FS, Crisponi L, de Geus EJ, Dehghan A, Deloukas P, Doney AS, Elliott P, Freimer N, Gateva V, Herder C, Hofman A, Hughes TE, Hunt S, Illig T, Inouye M, Isomaa B, Johnson T, Kong A, Krestyaninova M, Kuusisto J, Laakso M, Lim N, Lindblad U, Lindgren CM, McCann OT, Mohlke KL, Morris AD, Naitza S, Orru M, Palmer CN, Pouta A, Randall J, Rathmann W, Saramies J, Scheet P, Scott LJ, Scuteri A, Sharp S, Sijbrands E, Smit JH, Song K, Steinthorsdottir V, Stringham HM, Tuomi T, Tuomilehto J, Uitterlinden AG, Voight BF, Waterworth D, Wichmann HE, Willemsen G, Witteman JC, Yuan X, Zhao JH, Zeggini E, Schlessinger D, Sandhu M, Boomsma DI, Uda M, Spector TD, Penninx BW, Altshuler D, Vollenweider P, Jarvelin MR, Lakatta E, Waeber G, Fox CS, Peltonen L, Groop LC, Mooser V, Cupples LA, Thorsteinsdottir U, Boehnke M, Barroso I, Van Duijn C, Dupuis J, Watanabe RM, Stefansson K, McCarthy MI, Wareham NJ, Meigs JB, Abecasis GR: Variants in MTNR1B influence fasting glucose levels. *Nat Genet* 41:77-81, 2009

19. Winckler W, Weedon MN, Graham RR, McCarroll SA, Purcell S, Almgren P, Tuomi T, Gaudet D, Bostrom KB, Walker M, Hitman G, Hattersley AT, McCarthy MI, Ardlie KG, Hirschhorn JN, Daly MJ, Frayling TM, Groop L, Altshuler D: Evaluation of common variants in the six known maturity-onset diabetes of the young (MODY) genes for association with type 2 diabetes. *Diabetes* 56:685-693, 2007
