# Supplementary material for: A Genome-Wide Association Search for Type 2 Diabetes Genes in African Americans
Source: PLoS One. 2012 Jan 4;7(1):e29202. doi: 10.1371/journal.pone.0029202 (PMC3251563; doi:10.1371/journal.pone.0029202)
Supplement: Table S3 — Validation P-values for T2DM loci across the genome. SNPs are ordered by chromosome and position (NCBI Build 36.1, hg18) with the major/minor alleles (positive strand) and corresponding gene (underlined) or nearest annotated gene. For the T2DM, IRAS and IRASFS analyses, the minor allele frequency (MAF) for T2DM and control populations are listed with the corresponding additive P-value. Note: For IRASFS MAFs are derived from the overall sample including relatives. For the Validation meta-analysis the additive P-value and odds ratio (OR) with associated 95% confidence interval (CI) are presented with respect to the minor allele. (DOC) [file pone.0029202.s005.doc]

**Supplementary Table 3. Validation *P-values* for T2DM loci across the genome.** SNPs are ordered by chromosome and position (NCBI Build 36.1, hg18) with the major/minor alleles (positive strand) and corresponding gene (underlined) or nearest annotated gene. For the T2DM, IRAS and IRASFS analyses, the minor allele frequency (MAF) for T2DM and control populations are listed with the corresponding additive *P-value*. Note: For IRASFS MAFs are derived from the overall sample including relatives. For the Validation meta-analysis the additive *P-value* and odds ratio (OR) with associated 95% confidence interval (CI) are presented with respect to the minor allele.

| **Locus** | | | | |  | **T2DM** | | |  | **IRAS** | | |  | **IRASFS** | | |  | **Meta-Analysis** | | |
| --- | --- | --- | --- | --- | --- | --- | --- | --- | --- | --- | --- | --- | --- | --- | --- | --- | --- | --- | --- | --- |
|  |  |  |  |  |  | **T2DM (n=1,246)** | | |  | **T2DM (n=115)** | | |  | **T2DM (n=97)** | | |  | **T2DM (n=1,458)** | | |
|  |  |  |  |  |  | **Control (n=927)** | | |  | **Control (n=164)** | | |  | **Control (n=507)** | | |  | **Control (n=1,598)** | | |
| **SNP** | **Chr** | **Position** | **Alleles** | **Nearest Gene(s)** |  | **MAF T2DM** | **MAF Control** | **Additive**  **P-Value** |  | **MAF T2DM** | **MAF Control** | **Additive**  **P-Value** |  | **MAF T2DM** | **MAF Control** | **Additive**  **P-Value** |  | **Additive**  **P-Value** | **OR (95% CI)** | **Het**  **P-Value** |
| rs7542900 | 1 | 94842629 | C/T | *F3 / SLC44A3* |  | 0.38 | 0.43 | **0.0033** |  | 0.39 | 0.43 | 0.31 |  | 0.40 | 0.42 | 0.58 |  | **2.4E-03** | 0.86 (0.78-0.96) | 0.68 |
| rs4659485 | 1 | 235212541 | T/C | *MTR / RYR2* |  | 0.08 | 0.11 | **0.0019** |  | 0.12 | 0.17 | 0.29 |  | 0.15 | 0.13 | 0.92 |  | **2.6E-03** | 0.78 (0.65-0.93) | 0.42 |
| rs7560163 | 2 | 151346182 | C/G | *RND3 / RBM43* |  | 0.11 | 0.16 | **3.7E-06** |  | 0.13 | 0.11 | 0.80 |  | 0.08 | 0.14 | **0.031** |  | **1.8E-06** | 0.74 (0.63-0.87) | 0.19 |
| rs2722769 | 11 | 11184950 | C/G | *ZBED5 / GALNTL4* |  | 0.07 | 0.09 | **0.022** |  | 0.10 | 0.12 | 0.80 |  | 0.04 | 0.08 | *0.068* |  | **4.9E-03** | 0.78 (0.65-0.94) | 0.71 |
| rs7107217 | 11 | 128978900 | C/A | *BARX2 / NFRKB* |  | 0.44 | 0.48 | **0.016** |  | 0.49 | 0.46 | 0.64 |  | 0.34 | 0.41 | 0.15 |  | **0.011** | 0.90 (0.81-1.00) | 0.43 |
