# Supplementary material for: A Genome-Wide Association Search for Type 2 Diabetes Genes in African Americans
Source: PLoS One. 2012 Jan 4;7(1):e29202. doi: 10.1371/journal.pone.0029202 (PMC3251563; doi:10.1371/journal.pone.0029202)
Supplement: Table S4 — Association results for African-American T2DM loci in the Diabetes Genetics Replication and Meta-analysis (DIAGRAM) Consortium. SNPs are ordered by chromosome and position (NCBI Build 36.1, hg18) and the nearest annotated gene is listed. For each SNP the major/minor alleles identified in the Overall African-American meta-analysis are indexed on the forward strand. Results from the association analysis in the Overall African-American cohort and DIAGRAM Consortium include the allele frequency (AF), odds ratio (OR) with associated 95% confidence interval (CI) and P-value with respect to the minor allele identified in the African-American population. SNP rs7560163 did not pass quality control filters in the DIAGRAM Consortium and was not included in analysis. (DOC) [file pone.0029202.s006.doc]

**Supplementary Table 4. Association results for African-American T2DM loci in the Diabetes Genetics Replication and Meta-analysis (DIAGRAM) Consortium.** SNPs are ordered by chromosome and position (NCBI Build 36.1, hg18) and the nearest annotated gene is listed. For each SNP the major/minor alleles identified in the Overall African-American meta-analysis are indexed on the forward strand. Results from the association analysis in the Overall African-American cohort and DIAGRAM Consortium include the allele frequency (AF), odds ratio (OR) with associated 95% confidence interval (CI) and *P-value* with respect to the minor allele identified in the African-American population. SNP rs7560163 did not pass quality control filters in the DIAGRAM Consortium and was not included in analysis.

|  |  |  |  |  |  | **Overall** | | |  | **DIAGRAM Consortium** | | |
| --- | --- | --- | --- | --- | --- | --- | --- | --- | --- | --- | --- | --- |
|  |  |  |  |  |  | **T2DM-ESRD + T2DM (n=3,132)** | | |  | **(Effective Sample Size = 22,570)** | | |
|  |  |  |  |  |  | **Controls (n=3,317)** | | |  |
| **SNP** | **Chr** | **Position** | **Nearest Gene(s)** | **Alleles** |  | **AF** | **OR (95% CI)** | **P-value** |  | **AF** | **OR (95% CI)** | **P-value** |
| rs7542900 | 1 | 94842629 | *F3 / SLC44A3* | C/T |  | 0.44 | 0.86 (0.80-0.92) | 6.0E-06 |  | 0.20 | 0.97 (0.92-1.02) | 0.26 |
| rs4659485 | 1 | 235212541 | *MTR / RYR2* | T/C |  | 0.11 | 0.77 (0.68-0.87) | 1.9E-05 |  | 0.52 | 1.01 (0.97-1.05) | 0.54 |
| rs7560163 | 2 | 151346182 | *RND3 / RBM43* | C/G |  | 0.14 | 0.75 (067-0.84) | 7.0E-09 |  | 0.01 | - | - |
| rs2722769 | 11 | 11184950 | *ZBED5 / GALNTL4* | C/G |  | 0.47 | 0.74 (0.65-0.84) | 1.7E-06 |  | 0.44 | 0.97 (0.93-1.01) | 0.19 |
| rs7107217 | 11 | 128978900 | *BARX2 / NFRKB* | C/A |  | 0.09 | 0.85 (0.79-0.91) | 3.2E-07 |  | 0.50 | 0.97 (0.93-1.01) | *0.086* |
