# Supplementary material for: A Genome-Wide Association Search for Type 2 Diabetes Genes in African Americans
Source: PLoS One. 2012 Jan 4;7(1):e29202. doi: 10.1371/journal.pone.0029202 (PMC3251563; doi:10.1371/journal.pone.0029202)
Supplement: Table S5 — Quantitative trait meta-analysis for African-American T2DM loci across the genome. SNPs are ordered by chromosome and position (NCBI Build 36.1, hg18) with the major/minor alleles (positive strand) and the nearest annotated gene is listed. For the IRAS and IRASFS samples, the β coefficient with respect to the minor allele is listed with the corresponding additive P-value. For the meta-analysis, the z-statistics is listed with the corresponding additive P-value. (DOC) [file pone.0029202.s007.doc]

**Supplementary Table 5. Quantitative trait meta-analysis for African-American T2DM loci across the genome. SNPs are ordered by chromosome and position (NCBI Build 36.1, hg18) with the major/minor alleles (positive strand) and the nearest annotated gene is listed. For the IRAS and IRASFS samples, the β coefficient with respect to the minor allele is listed with the corresponding additive *P-value.* For the meta-analysis, the z-statistics is listed with the corresponding additive *P-value*.**

|  |  |  |  |  | **IRAS** | | **IRASFS** | | **Meta-Analysis** | |
| --- | --- | --- | --- | --- | --- | --- | --- | --- | --- | --- |
|  |  |  |  |  | **Control (n=164)** | | **Control (n=507)** | | **Control (n=671)** | |
| **SNP** | **Chr** | **Position** | **Alleles** | **Nearest Gene(s)** | **β** | **Additive**  **P-Value** | **β** | **Additive**  **P-Value** | **Z** | **Additive**  **P-Value** |
| ***Insulin Sensitivity (S_I_)*** | | | | | | | | | | |
| rs7542900 | 1 | 94842629 | C/T | *F3 / SLC44A3* | -0.06 | 0.25 | 0.01 | 0.55 | -0.05 | 0.96 |
| rs4659485 | 1 | 235212541 | T/C | *MTR / RYR2* | -0.04 | 0.66 | 0.01 | 0.79 | -0.03 | 0.98 |
| rs7560163 | 2 | 151346182 | C/G | *RND3 / RBM43* | -0.09 | 0.22 | -0.04 | 0.22 | 1.67 | *0.094* |
| rs2722769 | 11 | 11184950 | C/G | *ZBED5 / GALNTL4* | -0.08 | 0.39 | -0.06 | 0.16 | 1.65 | 0.10 |
| rs7107217 | 11 | 128978900 | C/A | *BARX2 / NFRKB* | -0.04 | 0.43 | -0.01 | 0.67 | -0.77 | 0.44 |
| ***Glucose Effectiveness (S_G_)*** | | | | | | | | | | |
| rs7542900 | 1 | 94842629 | C/T | *F3 / SLC44A3* | 0.00 | 0.75 | 0.00 | 0.91 | -0.06 | 0.96 |
| rs4659485 | 1 | 235212541 | T/C | *MTR / RYR2* | 0.00 | 0.39 | 0.00 | 0.87 | -0.26 | 0.80 |
| rs7560163 | 2 | 151346182 | C/G | *RND3 / RBM43* | 0.00 | 0.19 | 0.00 | 0.27 | -0.31 | 0.75 |
| rs2722769 | 11 | 11184950 | C/G | *ZBED5 / GALNTL4* | 0.00 | 0.46 | 0.00 | 0.93 | 0.44 | 0.66 |
| rs7107217 | 11 | 128978900 | C/A | *BARX2 / NFRKB* | 0.00 | 0.14 | 0.00 | 0.22 | 1.78 | *0.075* |
| ***Acute Insulin Response (AIR)*** | | | | | | | | | | |
| rs7542900 | 1 | 94842629 | C/T | *F3 / SLC44A3* | 0.10 | 0.24 | 0.34 | 0.67 | 0.96 | 0.34 |
| rs4659485 | 1 | 235212541 | T/C | *MTR / RYR2* | 0.18 | 0.22 | -1.23 | 0.32 | 0.30 | 0.76 |
| rs7560163 | 2 | 151346182 | C/G | *RND3 / RBM43* | -0.13 | 0.33 | 1.67 | 0.15 | -0.76 | 0.45 |
| rs2722769 | 11 | 11184950 | C/G | *ZBED5 / GALNTL4* | 0.11 | 0.45 | -0.69 | 0.63 | 0.05 | 0.96 |
| rs7107217 | 11 | 128978900 | C/A | *BARX2 / NFRKB* | 0.06 | 0.46 | -0.01 | 0.99 | 0.35 | 0.73 |
| ***Disposition Index (DI)*** | | | | | | | | | | |
| rs7542900 | 1 | 94842629 | C/T | *F3 / SLC44A3* | -0.10 | 0.41 | 0.36 | 0.73 | -0.11 | 0.91 |
| rs4659485 | 1 | 235212541 | T/C | *MTR / RYR2* | 0.05 | 0.81 | -0.67 | 0.68 | 0.26 | 0.80 |
| rs7560163 | 2 | 151346182 | C/G | *RND3 / RBM43* | -0.34 | *0.058* | 0.92 | 0.55 | 0.42 | 0.67 |
| rs2722769 | 11 | 11184950 | C/G | *ZBED5 / GALNTL4* | -0.06 | 0.79 | -2.93 | 0.13 | 1.47 | 0.14 |
| rs7107217 | 11 | 128978900 | C/A | *BARX2 / NFRKB* | -0.10 | 0.39 | -0.33 | 0.75 | -0.69 | 0.49 |
| ***Fasting Glucose*** | | | | | | | | | | |
| rs7542900 | 1 | 94842629 | C/T | *F3 / SLC44A3* | 0.70 | 0.52 | -0.29 | 0.61 | -0.10 | 0.92 |
| rs4659485 | 1 | 235212541 | T/C | *MTR / RYR2* | -2.79 | 0.14 | 1.00 | 0.29 | -0.18 | 0.86 |
| rs7560163 | 2 | 151346182 | C/G | *RND3 / RBM43* | 1.19 | 0.48 | -0.25 | 0.78 | -0.13 | 0.90 |
| rs2722769 | 11 | 11184950 | C/G | *ZBED5 / GALNTL4* | -2.58 | 0.15 | -0.04 | 0.97 | 0.77 | 0.44 |
| rs7107217 | 11 | 128978900 | C/A | *BARX2 / NFRKB* | 0.41 | 0.71 | -0.32 | 0.58 | -0.28 | 0.78 |
| ***Fasting Insulin*** | | | | | | | | | | |
| rs7542900 | 1 | 94842629 | C/T | *F3 / SLC44A3* | 0.07 | 0.16 | -0.05 | 0.23 | -0.29 | 0.77 |
| rs4659485 | 1 | 235212541 | T/C | *MTR / RYR2* | -0.02 | 0.83 | -0.05 | 0.40 | 0.83 | 0.41 |
| rs7560163 | 2 | 151346182 | C/G | *RND3 / RBM43* | -0.01 | 0.88 | 0.07 | 0.27 | -0.87 | 0.39 |
| rs2722769 | 11 | 11184950 | C/G | *ZBED5 / GALNTL4* | 0.03 | 0.73 | 0.04 | 0.62 | -0.60 | 0.55 |
| rs7107217 | 11 | 128978900 | C/A | *BARX2 / NFRKB* | 0.08 | 0.11 | 0.08 | **0.044** | 2.54 | **0.011** |
