# Supplementary material for: A Genome-Wide Association Search for Type 2 Diabetes Genes in African Americans
Source: PLoS One. 2012 Jan 4;7(1):e29202. doi: 10.1371/journal.pone.0029202 (PMC3251563; doi:10.1371/journal.pone.0029202)
Supplement: Table S6 — Association results for African-American T2DM loci in the Meta-Analyses of Glucose and Insulin-related traits Consortium (MAGIC). SNPs are ordered by chromosome and position (NCBI Build 36.1, hg18) with the alleles on the positive strand (African American risk alleles are underlined) and the nearest annotated gene is listed. For each SNP and trait combination, the effect size and standard error are listed with the corresponding P-value. (DOC) [file pone.0029202.s008.doc]

**Supplementary Table 6. Association results for African-American T2DM loci in the** Meta-Analyses **of Glucose and Insulin-related traits Consortium (MAGIC).** SNPs are ordered by chromosome and position (NCBI Build 36.1, hg18) with the alleles on the positive strand (African American risk alleles are underlined) and the nearest annotated gene is listed. For each SNP and trait combination, the effect size and standard error are listed with the corresponding *P-value.*

| **SNP** | **Chr** | **Position** | **Alleles (1/2)** | **Allele 1 Frequency** | **Nearest Gene(s)** | **β ± SE** | **Global P-value** | **Joint Analysis N** |
| --- | --- | --- | --- | --- | --- | --- | --- | --- |
| ***Fasting Glucose (FGlu)*** | | |  |  |  |  |  |  |
| rs7542900 | 1 | 94842629 | T/C | 0.18 | *F3 / SLC44A3* | 0.0057 ± 0.0049 | 0.25 | 45,061 |
| rs4659485 | 1 | 235212541 | T/C | 0.48 | *MTR / RYR2* | 0.0021 ± 0.0036 | 0.55 | 46,159 |
| rs7560163 | 2 | 151346182 |  |  | *RND3 / RBM43* |  |  |  |
| rs2722769 | 11 | 11184950 | C/G | 0.59 | *ZBED5 / GALNTL4* | 0.0000 ± 0.0037 | 1.00 | 46,183 |
| rs7107217 | 11 | 128978900 | A/C | 0.47 | *BARX2 / NFRKB* | 0.0026 ± 0.0037 | 0.47 | 46,131 |
| ***Fasting Insulin (FINS)*** | | |  |  |  |  |  |  |
| rs7542900 | 1 | 94842629 | T/C | 0.18 | *F3 / SLC44A3* | 0.0008 ± 0.0052 | 0.88 | 37,211 |
| rs4659485 | 1 | 235212541 | T/C | 0.48 | *MTR / RYR2* | -0.0007 ± 0.0038 | 0.86 | 38,212 |
| rs7560163 | 2 | 151346182 |  |  | *RND3 / RBM43* |  |  |  |
| rs2722769 | 11 | 11184950 | C/G | 0.60 | *ZBED5 / GALNTL4* | 0.0059 ± 0.0039 | 0.13 | 38,235 |
| rs7107217 | 11 | 128978900 | A/C | 0.46 | *BARX2 / NFRKB* | 0.0043 ± 0.0038 | 0.26 | 38,184 |
| ***Homeostasis Model Assessment of Beta-cell Function (HOMA-B)*** | | | | | |  |  |  |
| rs7542900 | 1 | 94842629 | T/C | 0.17 | *F3 / SLC44A3* | -0.0007 ± 0.0045 | 0.88 | 35,447 |
| rs4659485 | 1 | 235212541 | T/C | 0.48 | *MTR / RYR2* | 0.0005 ± 0.0033 | 0.88 | 36,440 |
| rs7560163 | 2 | 151346182 |  |  | *RND3 / RBM43* |  |  |  |
| rs2722769 | 11 | 11184950 | C/G | 0.60 | *ZBED5 / GALNTL4* | 0.008 ± 0.0034 | **0.018** | 36,463 |
| rs7107217 | 11 | 128978900 | A/C | 0.48 | *BARX2 / NFRKB* | 0.0053 ± 0.0033 | 0.10 | 36,412 |
| ***Homeostasis Model Assessment of Insulin Resistance (HOMA-IR)*** | | | | | |  |  |  |
| rs7542900 | 1 | 94842629 | T/C | 0.18 | *F3 / SLC44A3* | 0.0017 ± 0.0054 | 0.75 | 35,913 |
| rs4659485 | 1 | 235212541 | T/C | 0.48 | *MTR / RYR2* | 0.0008 ± 0.0040 | 0.83 | 37,011 |
| rs7560163 | 2 | 151346182 |  |  | *RND3 / RBM43* |  |  |  |
| rs2722769 | 11 | 11184950 | C/G | 0.60 | *ZBED5 / GALNTL4* | 0.0094 ± 0.0041 | **0.022** | 37,034 |
| rs7107217 | 11 | 128978900 | A/C | 0.46 | *BARX2 / NFRKB* | 0.0051 ± 0.0040 | 0.20 | 36,983 |
| ***2 Hour Glucose adjusted for BMI*** | | | | | | | | |
| rs7542900 | 1 | 94842629 | T/C |  | *F3 / SLC44A3* | -0.0040 ± 0.025 | 0.87 | 15,234 |
| rs4659485 | 1 | 235212541 | T/C |  | *MTR / RYR2* | -0.0018 ± 0.019 | 0.92 | 15,228 |
| rs7560163 | 2 | 151346182 |  |  | *RND3 / RBM43* |  |  |  |
| rs2722769 | 11 | 11184950 | C/G |  | *ZBED5 / GALNTL4* | 0.023 ± 0.019 | 0.23 | 15,231 |
| rs7107217 | 11 | 128978900 | A/C |  | *BARX2 / NFRKB* | 0.014 ± 0.019 | 0.47 | 15,227 |
| ***2 Hour Insulin adjusted for BMI*** | | | | | | | | |
| rs7542900 | 1 | 94842629 | T/C |  | *F3 / SLC44A3* | -0.0075 ± 0.015 | 0.62 | 7,062 |
| rs4659485 | 1 | 235212541 | T/C |  | *MTR / RYR2* | 0.010 ± 0.011 | 0.36 | 7,056 |
| rs7560163 | 2 | 151346182 |  |  | *RND3 / RBM43* |  |  |  |
| rs2722769 | 11 | 11184950 | C/G |  | *ZBED5 / GALNTL4* | 0.0074 ± 0.012 | 0.53 | 7,059 |
| rs7107217 | 11 | 128978900 | A/C |  | *BARX2 / NFRKB* | 0.0031 ±0.011 | 0.78 | 7,056 |
| *HbA1c* | | | | | | | | |
| rs7542900 | 1 | 94842629 | T/C | 0.22 | *F3 / SLC44A3* | -0.0013 ± 0.0045 | 0.78 | 35,580 |
| rs4659485 | 1 | 235212541 | T/C | 0.49 | *MTR / RYR2* | 0.0020 ± 0.0034 | 0.56 | 35,819 |
| rs7560163 | 2 | 151346182 |  |  | *RND3 / RBM43* |  |  |  |
| rs2722769 | 11 | 11184950 | C/G | 0.58 | *ZBED5 / GALNTL4* | -0.0022 ± 0.0035 | 0.52 | 35,858 |
| rs7107217 | 11 | 128978900 | A/C | 0.46 | *BARX2 / NFRKB* | 0.0052 ± 0.0034 | 0.13 | 35,772 |
