# Supplementary material for: A Genome-Wide Association Search for Type 2 Diabetes Genes in African Americans
Source: PLoS One. 2012 Jan 4;7(1):e29202. doi: 10.1371/journal.pone.0029202 (PMC3251563; doi:10.1371/journal.pone.0029202)
Supplement: Table S7 — Gender stratified association analysis with T2DM. SNPs are ordered by chromosome and position (NCBI Build 36.1, hg18) with the major/minor alleles (positive strand) and corresponding gene (underlined) or nearest annotated genes (+/−500 kb). For males and females, the additive P-value and odds ratio (OR) with associated 95% confidence interval (CI) with respect to the minor allele and heterozygosity P-value are listed. (DOC) [file pone.0029202.s009.doc]

**Supplementary Table 7. Gender stratified association analysis with T2DM.** SNPs are ordered by chromosome and position (NCBI Build 36.1, hg18) with the major/minor alleles (positive strand) and corresponding gene (underlined) or nearest annotated genes (+/-500kb). For males and females, the additive *P*-value and odds ratio (OR) with associated 95% confidence interval (CI) with respect to the minor allele and heterozygosity P-value are listed.

| **Locus** | | | | **Overall - Males** | | |  | **Overall - Females** | | |
| --- | --- | --- | --- | --- | --- | --- | --- | --- | --- | --- |
|  |  |  |  | **T2DM-ESRD + T2DM (n_max_=2608)** | | |  | **T2DM-ESRD + T2DM (n_max_=3730)** | | |
|  |  |  |  | **Controls (n=3,317)** | | |  | **Controls (n=3,317)** | | |
| **SNP** | **Position** | **Alleles** | **Nearest Gene(s)** | **Additive P-Value** | **OR** | **Het**  **P-Value** |  | **Additive P-Value** | **OR** | **Het**  **P-Value** |
|  |  |  |  |  | **(95% CI)** |  |  |  | **(95% CI)** |  |
| rs7542900 | Chr1:94842629 | C/T | *SLC44A3*  *F3* | **9.5E-04** | 0.83 | 0.61 |  | **0.0059** | 0.90 | 0.40 |
|  |  |  |  |  | 0.74-0.93 |  |  |  | 0.82-0.98 |  |
| rs4659485 | Chr1:235212541 | T/C | *RYR2*  *MTR* | *0.072* | 0.83 | 0.62 |  | **9.5E-05** | 0.74 | 0.59 |
|  |  |  |  |  | 0.69-1.01 |  |  |  | 0.63-0.88 |  |
| rs7560163 | Chr2:151346182 | C/G | *RBM43*  *RND3* | **0.0022** | 0.80 | 0.13 |  | **3.2E-07** | 0.72 | 0.64 |
|  |  |  |  |  | 0.67-0.95 |  |  |  | 0.62-0.83 |  |
| rs3775045 | Chr4:96345907 | C/T | *UNC5C* | **0.028** | 1.13 | 0.49 |  | **1.5E-04** | 1.20 | 0.23 |
|  |  |  |  |  | 1.00-1.27 |  |  |  | 1.08-1.33 |  |
| rs6451146 | Chr5:34599780 | T/C | *RAI14*  *-* | **0.0056** | 0.83 | 0.26 |  | **9.5E-04** | 0.79 | 0.56 |
|  |  |  |  |  | 0.71-0.98 |  |  |  | 0.69-0.90 |  |
| rs6930576 | Chr6:148746647 | G/A | *SASH1* | **4.6E-04** | 1.24 | 0.23 |  | **0.0082** | 1.13 | 0.34 |
|  |  |  |  |  | 1.09-1.40 |  |  |  | 1.02-1.26 |  |
| rs17103805 | Chr10:86418457 | A/G | *-*  *FAM190B* | **0.0062** | 1.23 | **0.042** |  | **0.0010** | 1.25 | 0.49 |
|  |  |  |  |  | 1.03-1.46 |  |  |  | 1.09-1.44 |  |
| rs2722769 | Chr11:11184950 | C/G | *GALNTL4*  *LOC729013* | **0.0037** | 0.76 | 0.79 |  | **5.7E-05** | 0.70 | 0.80 |
|  |  |  |  |  | 0.62-0.94 |  |  |  | 0.59-0.84 |  |
| rs7107217 | Chr11:128978900 | C/A | *TMEM45B*  *BARX2* | **3.9E-06** | 0.79 | 0.27 |  | **0.0030** | 0.88 | 0.91 |
|  |  |  |  |  | 0.71-0.89 |  |  |  | 0.80-0.97 |  |
| rs1271784 | Chr18:30972595 | A/T | *MAPRE2* | **0.0090** | 1.14 | 0.22 |  | **6.7E-04** | 1.21 | 0.29 |
|  |  |  |  |  | 0.99-1.32 |  |  |  | 1.08-1.35 |  |
