# Supplementary material for: A Genome-Wide Association Search for Type 2 Diabetes Genes in African Americans
Source: PLoS One. 2012 Jan 4;7(1):e29202. doi: 10.1371/journal.pone.0029202 (PMC3251563; doi:10.1371/journal.pone.0029202)
Supplement: Table S8 — Power Calculations. Table S8a. Genome-wide association study power analysis for causal variant in complete and incomplete linkage disequilibrium with a typed variant given minor allele frequency (p) in 965 cases and 1029 controls. Table S8b. Replication power analysis for causal variant in complete and incomplete linkage disequilibrium with a typed variant given minor allele frequency (p) in 709 cases and 690 controls. Table S8c. GWAS + Replication sample power analysis for causal variant in complete and incomplete linkage disequilibrium with a typed variant given minor allele frequency (p) in 1674 cases and 1719 controls. Table S8d. T2DM power analysis for causal variant in complete and incomplete linkage disequilibrium with a typed variant given minor allele frequency (p) in 1246 cases and 927 controls. Table S8e. IRAS power analysis for causal variant in complete and incomplete linkage disequilibrium with a typed variant given minor allele frequency (p) in 115 cases and 164 controls. Table S8f. IRASFS power analysis for causal variant in complete and incomplete linkage disequilibrium with a typed variant given minor allele frequency (p) in 97 cases and 507 controls. Table S8g. Validation meta-analysis power analysis for causal variant in complete and incomplete linkage disequilibrium with a typed variant given minor allele frequency (p) in 1458 cases and 1598 controls. Table S8h. Overall power analysis for causal variant in complete and incomplete linkage disequilibrium with a typed variant given minor allele frequency (p) in 3132 cases and 3317 controls. (DOC) [file pone.0029202.s010.doc]

**Supplementary Table 8. Power Calculations**

Supplementary Table 8a. Genome-wide association study power analysis for causal variant in complete and incomplete linkage disequilibrium with a typed variant given minor allele frequency (p) in 965 cases and 1029 controls.

|  | | Detectable Odds Ratio (Risk and [Protection]) under an Additive Model | | | | | | | |
| --- | --- | --- | --- | --- | --- | --- | --- | --- | --- |
|  |  | Complete Linkage Disequilibrium (r^2^=1.00) | | | | High Linkage Disequilibrium (r^2^=0.80) | | | |
| Type 1 Error | Power | p=0.40 | p=0.30 | p=0.20 | p=0.10 | p=0.40 | p=0.30 | p=0.20 | p=0.10 |
| 0.05 | 0.7 | 1.17 [0.85] | 1.19 [0.84] | 1.21 [0.83] | 1.29 [0.78] | 1.20 [0.83] | 1.21 [0.83] | 1.24 [0.81] | 1.32 [0.76] |
|  | 0.8 | 1.19 [0.84] | 1.21 [0.83] | 1.24 [0.81] | 1.33 [0.75] | 1.22 [0.82] | 1.24 [0.81] | 1.27 [0.79] | 1.37 [0.73] |
|  | 0.9 | 1.23 [0.81] | 1.25 [0.80] | 1.28 [0.78] | 1.38 [0.72] | 1.26 [0.79] | 1.28 [0.78] | 1.32 [0.76] | 1.43 [0.70] |
| 0.001 | 0.7 | 1.28 [0.78] | 1.30 [0.77] | 1.34 [0.75] | 1.46 [0.68] | 1.32 [0.76] | 1.33 [0.75] | 1.38 [0.72] | 1.51 [0.66] |
|  | 0.8 | 1.30 [0.77] | 1.32 [0.76] | 1.37 [0.73] | 1.50 [0.67] | 1.35 [0.74] | 1.37 [0.73] | 1.42 [0.70] | 1.57 [0.64] |
|  | 0.9 | 1.34 [0.75] | 1.36 [0.74] | 1.42 [0.70] | 1.56 [0.64] | 1.39 [0.72] | 1.41 [0.71] | 1.47 [0.68] | 1.64 [0.61] |
| 5.0E-7 | 0.7 | 1.43 [0.70] | 1.45 [0.69] | 1.52 [0.66] | 1.70 [0.59] | 1.49 [0.67] | 1.52 [0.66] | 1.59 [0.63] | 1.80 [0.56] |
|  | 0.8 | 1.46 [0.68] | 1.48 [0.68] | 1.55 [0.65] | 1.75 [0.57] | 1.52 [0.66] | 1.55 [0.65] | 1.63 [0.61] | 1.85 [0.54] |
|  | 0.9 | 1.50 [0.67] | 1.53 [0.65] | 1.60 [0.63] | 1.82 [0.55] | 1.57 [0.64] | 1.60 [0.63] | 1.69 [0.59] | 1.94 [0.52] |

Supplementary Table 8b. Replication power analysis for causal variant in complete and incomplete linkage disequilibrium with a typed variant given minor allele frequency (p) in 709 cases and 690 controls.

|  | | Detectable Odds Ratio (Risk and [Protection]) under an Additive Model | | | | | | | |
| --- | --- | --- | --- | --- | --- | --- | --- | --- | --- |
|  |  | Complete Linkage Disequilibrium (r^2^=1.00) | | | | High Linkage Disequilibrium (r^2^=0.80) | | | |
| Type 1 Error | Power | p=0.40 | p=0.30 | p=0.20 | p=0.10 | p=0.40 | p=0.30 | p=0.20 | p=0.10 |
| 0.05 | 0.7 | 1.21 [0.83] | 1.23 [0.81] | 1.26 [0.79] | 1.35 [0.74] | 1.24 [0.81] | 1.25 [0.80] | 1.29 [0.78] | 1.39 [0.72] |
|  | 0.8 | 1.24 [0.81] | 1.26 [0.79] | 1.29 [0.78] | 1.39 [0.72] | 1.27 [0.79] | 1.29 [0.78] | 1.33 [0.75] | 1.45 [0.69] |
|  | 0.9 | 1.29 [0.78] | 1.30 [0.77] | 1.35 [0.74] | 1.47 [0.68] | 1.32 [0.76] | 1.34 [0.75] | 1.39 [0.72] | 1.53 [0.65] |
| 0.001 | 0.7 | 1.34 [0.75] | 1.36 [0.74] | 1.41 [0.71] | 1.56 [0.64] | 1.39 [0.72] | 1.41 [0.71] | 1.47 [0.68] | 1.64 [0.61] |
|  | 0.8 | 1.37 [0.73] | 1.39 [0.72] | 1.45 [0.69] | 1.61 [0.62] | 1.43 [0.70] | 1.45 [0.69] | 1.52 [0.66] | 1.70 [0.59] |
|  | 0.9 | 1.42 [0.70] | 1.44 [0.69] | 1.51 [0.66] | 1.69 [0.59] | 1.48 [0.68] | 1.51 [0.66] | 1.58 [0.63] | 1.79 [0.56] |
| 5.0E-7 | 0.7 | 1.53 [0.65] | 1.56 [0.64] | 1.64 [0.61] | 1.87 [0.53] | 1.60 [0.63] | 1.64 [0.61] | 1.73 [0.58] | 2.00 [0.50] |
|  | 0.8 | 1.57 [0.64] | 1.60 [0.63] | 1.68 [0.60] | 1.93 [0.52] | 1.65 [0.61] | 1.69 [0.59] | 1.79 [0.56] | 2.07 [0.48] |
|  | 0.9 | 1.62 [0.62] | 1.66 [0.60] | 1.75 [0.57] | 2.02 [0.50] | 0.72 [1.39] | 1.75 [0.57] | 1.86 [0.54] | 2.18 [0.46] |

Supplementary Table 8c. GWAS + Replication sample power analysis for causal variant in complete and incomplete linkage disequilibrium with a typed variant given minor allele frequency (p) in 1674 cases and 1719 controls.

|  | | Detectable Odds Ratio (Risk and [Protection]) under an Additive Model | | | | | | | |
| --- | --- | --- | --- | --- | --- | --- | --- | --- | --- |
|  |  | Complete Linkage Disequilibrium (r^2^=1.00) | | | | High Linkage Disequilibrium (r^2^=0.80) | | | |
| Type 1 Error | Power | p=0.40 | p=0.30 | p=0.20 | p=0.10 | p=0.40 | p=0.30 | p=0.20 | p=0.10 |
| 0.05 | 0.7 | 1.13 [0.88] | 1.14 [0.88] | 1.16 [0.86] | 1.22 [0.82] | 1.15 [0.87] | 1.16 [0.86] | 1.18 [0.85] | 1.24 [0.81] |
|  | 0.8 | 1.15 [0.87] | 1.16 [0.86] | 1.18 [0.85] | 1.24 [0.81] | 1.17 [0.85] | 1.18 [0.85] | 1.20 [0.83] | 1.27 [0.79] |
|  | 0.9 | 1.17 [0.85] | 1.19 [0.84] | 1.21 [0.83] | 1.29 [0.78] | 1.20 [0.83] | 1.21 [0.83] | 1.24 [0.81] | 1.32 [0.76] |
| 0.001 | 0.7 | 1.21 [0.83] | 1.22 [0.82] | 1.25 [0.80] | 1.34 [0.75] | 1.24 [0.81] | 1.25 [0.80] | 1.29 [0.78] | 1.39 [0.72] |
|  | 0.8 | 1.23 [0.81] | 1.24 [0.81] | 1.28 [0.78] | 1.37 [0.73] | 1.26 [0.79] | 1.27 [0.79] | 1.31 [0.76] | 1.42 [0.70] |
|  | 0.9 | 1.25 [0.80] | 1.27 [0.79] | 1.31 [0.76] | 1.41 [0.71] | 1.29 [0.78] | 1.31 [0.76] | 1.35 [0.74] | 1.47 [0.68] |
| 5.0E-7 | 0.7 | 1.32 [0.76] | 1.33 [0.75] | 1.38 [0.72] | 1.52 [0.66] | 1.36 [0.74] | 1.38 [0.72] | 1.43 [0.70] | 1.59 [0.63] |
|  | 0.8 | 1.34 [0.75] | 1.36 [0.74] | 1.41 [0.71] | 1.55 [0.65] | 1.38 [0.72] | 1.40 [0.71] | 1.46 [0.68] | 1.62 [0.62] |
|  | 0.9 | 1.36 [0.74] | 1.38 [0.72] | 1.44 [0.69] | 1.60 [0.63] | 1.42 [0.70] | 1.44 [0.69] | 1.50 [0.67] | 1.68 [0.60] |

Supplementary Table 8d. T2DM power analysis for causal variant in complete and incomplete linkage disequilibrium with a typed variant given minor allele frequency (p) in 1246 cases and 927 controls.

|  | | Detectable Odds Ratio (Risk and [Protection]) under an Additive Model | | | | | | | |
| --- | --- | --- | --- | --- | --- | --- | --- | --- | --- |
|  |  | Complete Linkage Disequilibrium (r^2^=1.00) | | | | High Linkage Disequilibrium (r^2^=0.80) | | | |
| Type 1 Error | Power | p=0.40 | p=0.30 | p=0.20 | p=0.10 | p=0.40 | p=0.30 | p=0.20 | p=0.10 |
| 0.05 | 0.7 | 1.17 [0.85] | 1.18 [0.85] | 1.21 [0.83] | 1.28 [0.78] | 1.19 [0.84] | 1.20 [0.83] | 1.23 [0.81] | 1.32 [0.76] |
|  | 0.8 | 1.19 [0.84] | 1.21 [0.83] | 1.24 [0.81] | 1.32 [0.76] | 1.22 [0.82] | 1.23 [0.81] | 1.26 [0.79] | 1.36 [0.74] |
|  | 0.9 | 1.22 [0.82] | 1.24 [0.81] | 1.28 [0.78] | 1.38 [0.72] | 1.25 [0.80] | 1.27 [0.79] | 1.31 [0.76] | 1.43 [0.70] |
| 0.001 | 0.7 | 1.27 [0.79] | 1.29 [0.78] | 1.33 [0.75] | 1.45 [0.69] | 1.31 [0.76] | 1.33 [0.75] | 1.38 [0.72] | 1.51 [0.66] |
|  | 0.8 | 1.30 [0.77] | 1.31 [0.76] | 1.36 [0.74] | 1.49 [0.67] | 1.34 [0.75] | 1.36 [0.74] | 1.41 [0.71] | 1.56 [0.64] |
|  | 0.9 | 1.33 [0.75] | 1.35 [0.74] | 1.41 [0.71] | 1.56 [0.64] | 1.38 [0.72] | 1.40 [0.71] | 1.46 [0.68] | 1.64 [0.61] |
| 5.0E-7 | 0.7 | 1.41 [0.71] | 1.44 [0.69] | 1.51 [0.66] | 1.70 [0.59] | 1.47 [0.68] | 1.50 [0.67] | 1.58 [0.63] | 1.81 [0.55] |
|  | 0.8 | 1.44 [0.69] | 1.47 [0.68] | 1.54 [0.65] | 1.75 [0.57] | 1.51 [0.66] | 1.54 [0.65] | 1.62 [0.62] | 1.87 [0.53] |
|  | 0.9 | 1.48 [0.68] | 1.51 [0.66] | 1.59 [0.63] | 1.83 [0.55] | 1.55 [0.65] | 1.59 [0.63] | 1.68 [0.60] | 1.96 [0.51] |

Supplementary Table 8e. IRAS power analysis for causal variant in complete and incomplete linkage disequilibrium with a typed variant given minor allele frequency (p) in 115 cases and 164 controls.

|  | | Detectable Odds Ratio (Risk and [Protection]) under an Additive Model | | | | | | | |
| --- | --- | --- | --- | --- | --- | --- | --- | --- | --- |
|  |  | Complete Linkage Disequilibrium (r^2^=1.00) | | | | High Linkage Disequilibrium (r^2^=0.80) | | | |
| Type 1 Error | Power | p=0.40 | p=0.30 | p=0.20 | p=0.10 | p=0.40 | p=0.30 | p=0.20 | p=0.10 |
| 0.05 | 0.7 | 1.55 [0.65] | 1.58 [0.63] | 1.67 [0.60] | 1.93 [0.52] | 1.63 [0.61] | 1.67 [0.60] | 1.77 [0.56] | 2.07 [0.48] |
|  | 0.8 | 1.64 [0.61] | 1.68 [0.60] | 1.78 [0.56] | 2.08 [0.48] | 1.74 [0.57] | 1.78 [0.56] | 1.90 [0.53] | 2.26 [0.44] |
|  | 0.9 | 1.77 [0.56] | 1.82 [0.55] | 1.94 [0.52] | 2.32 [0.43] | 1.91 [0.52] | 1.95 [0.51] | 2.10 [0.48] | 2.53 [0.40] |
| 0.001 | 0.7 | 1.96 [0.51] | 2.02 [0.50] | 2.18 [0.46] | 2.68 [0.37] | 2.14 [0.47] | 2.20 [0.45] | 2.40 [0.42] | 3.00 [0.33] |
|  | 0.8 | 2.08 [0.48] | 2.14 [0.47] | 2.32 [0.43] | 2.90 [0.34] | 2.28 [0.44] | 2.36 [0.42] | 2.58 [0.39] | 3.28 [0.30] |
|  | 0.9 | 2.26 [0.44] | 2.34 [0.43] | 2.56 [0.39] | 3.24 [0.31] | 2.52 [0.40] | 2.60 [0.38] | 2.88 [0.35] | 3.72 [0.27] |
| 5.0E-7 | 0.7 | 2.74 [0.36] | 2.84 [0.35] | 3.14 [0.32] | 4.19 [0.24] | 3.14 [0.32] | 3.27 [0.31] | 3.66 [0.27] | 4.99 [0.20] |
|  | 0.8 | 2.93 [0.34] | 3.04 [0.33] | 3.38 [0.30] | 4.54 [0.22] | 3.40 [0.29] | 3.54 [0.28] | 3.98 [0.25] | 5.52 [0.18] |
|  | 0.9 | 3.22 [0.31] | 3.34 [0.30] | 3.74 [0.27] | 5.13 [0.19] | 3.80 [0.26] | 3.98 [0.25] | 4.50 [0.22] | 6.38 [0.16] |

Supplementary Table 8f. IRASFS power analysis for causal variant in complete and incomplete linkage disequilibrium with a typed variant given minor allele frequency (p) in 97 cases and 507 controls.

|  | | Detectable Odds Ratio (Risk and [Protection]) under an Additive Model | | | | | | | |
| --- | --- | --- | --- | --- | --- | --- | --- | --- | --- |
|  |  | Complete Linkage Disequilibrium (r^2^=1.00) | | | | High Linkage Disequilibrium (r^2^=0.80) | | | |
| Type 1 Error | Power | p=0.40 | p=0.30 | p=0.20 | p=0.10 | p=0.40 | p=0.30 | p=0.20 | p=0.10 |
| 0.05 | 0.7 | 1.48 [0.68] | 1.51 [0.66] | 1.59 [0.63] | 1.80 [0.56] | 1.56 [0.64] | 1.59 [0.63] | 1.67 [0.60] | 1.92 [0.52] |
|  | 0.8 | 1.56 [0.64] | 1.60 [0.63] | 1.68 [0.60] | 1.93 [0.52] | 1.64 [0.61] | 1.68 [0.60] | 1.78 [0.56] | 2.07 [0.48] |
|  | 0.9 | 1.68 [0.60] | 1.71 [0.58] | 1.82 [0.55] | 2.12 [0.47] | 1.78 [0.56] | 1.82 [0.55] | 1.94 [0.52] | 2.30 [0.43] |
| 0.001 | 0.7 | 1.84 [0.54] | 1.88 [0.53] | 2.01 [0.50] | 2.39 [0.42] | 1.98 [0.51] | 2.04 [0.49] | 2.18 [0.46] | 2.64 [0.38] |
|  | 0.8 | 1.94 [0.52] | 1.99 [0.50] | 2.13 [0.47] | 2.56 [0.39] | 2.10 [0.48] | 2.14 [0.47] | 2.32 [0.43] | 2.84 [0.35] |
|  | 0.9 | 2.09 [0.48] | 2.14 [0.47] | 2.30 [0.43] | 2.81 [0.36] | 2.28 [0.44] | 2.34 [0.43] | 2.54 [0.39] | 3.13 [0.32] |
| 5.0E-7 | 0.7 | 2.45 [0.41] | 2.51 [0.40] | 2.75 [0.36] | 3.46 [0.29] | 2.75 [0.36] | 2.84 [0.35] | 3.11 [0.32] | 4.01 [0.25] |
|  | 0.8 | 2.60 [0.38] | 2.66 [0.38] | 2.93 [0.34] | 3.74 [0.27] | 2.94 [0.34] | 3.02 [0.33] | 3.32 [0.30] | 4.34 [0.23] |
|  | 0.9 | 2.81 [0.36] | 2.90 [0.34] | 3.17 [0.32] | 4.13 [0.24] | 3.23 [0.31] | 3.32 [0.30] | 3.68 [0.27] | 4.88 [0.20] |

Supplementary Table 8g. Validation meta-analysis power analysis for causal variant in complete and incomplete linkage disequilibrium with a typed variant given minor allele frequency (p) in 1458 cases and 1598 controls.

|  | | Detectable Odds Ratio (Risk and [Protection]) under an Additive Model | | | | | | | |
| --- | --- | --- | --- | --- | --- | --- | --- | --- | --- |
|  |  | Complete Linkage Disequilibrium (r^2^=1.00) | | | | High Linkage Disequilibrium (r^2^=0.80) | | | |
| Type 1 Error | Power | p=0.40 | p=0.30 | p=0.20 | p=0.10 | p=0.40 | p=0.30 | p=0.20 | p=0.10 |
| 0.05 | 0.7 | 1.14 [0.88] | 1.15 [0.87] | 1.17 [0.85] | 1.23 [0.81] | 1.16 [0.86] | 1.17 [0.85] | 1.19 [0.84] | 1.26 [0.79] |
|  | 0.8 | 1.16 [0.86] | 1.17 [0.85] | 1.19 [0.84] | 1.26 [0.79] | 1.18 [0.85] | 1.19 [0.84] | 1.22 [0.82] | 1.29 [0.78] |
|  | 0.9 | 1.18 [0.85] | 1.20 [0.83] | 1.22 [0.82] | 1.30 [0.77] | 1.21 [0.83] | 1.22 [0.82] | 1.26 [0.79] | 1.34 [0.75] |
| 0.001 | 0.7 | 1.22 [0.82] | 1.24 [0.81] | 1.27 [0.79] | 1.36 [0.74] | 1.25 [0.80] | 1.27 [0.79] | 1.30 [0.77] | 1.41 [0.71] |
|  | 0.8 | 1.24 [0.81] | 1.26 [0.79] | 1.29 [0.78] | 1.39 [0.72] | 1.27 [0.79] | 1.29 [0.78] | 1.33 [0.75] | 1.45 [0.69] |
|  | 0.9 | 1.27 [0.79] | 1.28 [0.78] | 1.33 [0.75] | 1.44 [0.69] | 1.31 [0.76] | 1.32 [0.76] | 1.37 [0.73] | 1.50 [0.67] |
| 5.0E-7 | 0.7 | 1.34 [0.75] | 1.35 [0.74] | 1.41 [0.71] | 1.55 [0.65] | 1.38 [0.72] | 1.40 [0.71] | 1.46 [0.68] | 1.62 [0.62] |
|  | 0.8 | 1.36 [0.74] | 1.38 [0.72] | 1.43 [0.70] | 1.58 [0.63] | 1.41 [0.71] | 1.43 [0.70] | 1.49 [0.67] | 1.66 [0.60] |
|  | 0.9 | 1.39 [0.72] | 1.41 [0.71] | 1.47 [0.68] | 1.63 [0.61] | 1.44 [0.69] | 1.47 [0.68] | 1.53 [0.65] | 1.73 [0.58] |

Supplementary Table 8h. Overall power analysis for causal variant in complete and incomplete linkage disequilibrium with a typed variant given minor allele frequency (p) in 3132 cases and 3317 controls.

|  | | Detectable Odds Ratio (Risk and [Protection]) under an Additive Model | | | | | | | |
| --- | --- | --- | --- | --- | --- | --- | --- | --- | --- |
|  |  | Complete Linkage Disequilibrium (r^2^=1.00) | | | | High Linkage Disequilibrium (r^2^=0.80) | | | |
| Type 1 Error | Power | p=0.40 | p=0.30 | p=0.20 | p=0.10 | p=0.40 | p=0.30 | p=0.20 | p=0.10 |
| 0.05 | 0.7 | 1.09 [0.92] | 1.10 [0.91] | 1.12 [0.89] | 1.15 [0.87] | 1.10 [0.91] | 1.11 [0.90] | 1.13 [0.88] | 1.17 [0.85] |
|  | 0.8 | 1.11 [0.90] | 1.11 [0.90] | 1.13 [0.88] | 1.18 [0.85] | 1.12 [0.89] | 1.13 [0.88] | 1.15 [0.87] | 1.20 [0.83] |
|  | 0.9 | 1.13 [0.88] | 1.13 [0.88] | 1.15 [0.87] | 1.20 [0.83] | 1.14 [0.88] | 1.15 [0.87] | 1.17 [0.85] | 1.23 [0.81] |
| 0.001 | 0.7 | 1.15 [0.87] | 1.16 [0.86] | 1.18 [0.85] | 1.24 [0.81] | 1.17 [0.85] | 1.18 [0.85] | 1.20 [0.83] | 1.27 [0.79] |
|  | 0.8 | 1.16 [0.86] | 1.17 [0.85] | 1.19 [0.84] | 1.26 [0.79] | 1.18 [0.85] | 1.19 [0.84] | 1.22 [0.82] | 1.29 [0.78] |
|  | 0.9 | 1.18 [0.85] | 1.19 [0.84] | 1.22 [0.82] | 1.29 [0.78] | 1.20 [0.83] | 1.21 [0.83] | 1.25 [0.80] | 1.33 [0.75] |
| 5.0E-7 | 0.7 | 1.22 [0.82] | 1.23 [0.81] | 1.27 [0.79] | 1.36 [0.74] | 1.25 [0.80] | 1.26 [0.79] | 1.30 [0.77] | 1.41 [0.71] |
|  | 0.8 | 1.23 [0.81] | 1.25 [0.80] | 1.28 [0.78] | 1.38 [0.72] | 1.27 [0.79] | 1.28 [0.78] | 1.32 [0.76] | 1.43 [0.70] |
|  | 0.9 | 1.25 [0.80] | 1.27 [0.79] | 1.31 [0.76] | 1.42 [0.70] | 1.29 [0.78] | 1.30 [0.77] | 1.35 [0.74] | 1.47 [0.68] |
