# Supplementary material for: A Genome-Wide Association Search for Type 2 Diabetes Genes in African Americans
Source: PLoS One. 2012 Jan 4;7(1):e29202. doi: 10.1371/journal.pone.0029202 (PMC3251563; doi:10.1371/journal.pone.0029202)
Supplement: Table S9 — IRAS and IRASFS power analysis to detect a causal variant with the effect size observed in the T2DM cohort. (DOC) [file pone.0029202.s011.doc]

**Supplementary Table 9**. IRAS and IRASFS power analysis to detect a causal variant with the effect size observed in the T2DM cohort.

| **Locus** | | | |  | **T2DM** | | | | |  | **IRAS** | | | |  | **IRASFS** | | | |
| --- | --- | --- | --- | --- | --- | --- | --- | --- | --- | --- | --- | --- | --- | --- | --- | --- | --- | --- | --- |
|  |  |  |  |  | **T2DM (n=1,246)** | | | | |  | **T2DM (n=115)** | | | |  | **T2DM (n=97)** | | | |
|  |  |  |  |  | **Control (n=927)** | | | | |  | **Control (n=164)** | | | |  | **Control (n=507)** | | | |
|  |  |  |  |  | **MAF** | | **Ref** | **Additive** |  |  |  |  |  |  |  |  |  |  |  |
| **SNP** | **Chr** | **Position** | **Alleles** |  | **Case** | **Control** | **Allele** | **P-value** | **OR (95%CI)** |  | **α** | **Power** | **α** | **Power** |  | **α** | **Power** | **α** | **Power** |
| rs7542900 | 1 | 94842629 | C/T |  | 0.38 | 0.43 | T | **0.0033** | 0.83 (0.74-0.94) |  | 0.05 | 0.17 | 0.001 | 0.01 |  | 0.05 | 0.21 | 0.001 | 0.02 |
| rs4659485 | 1 | 235212541 | T/C |  | 0.08 | 0.11 | C | **0.0019** | 0.71 (0.57-0.88) |  |  | 0.22 |  | 0.02 |  |  | 0.25 |  | 0.02 |
| rs7560163 | 2 | 151346182 | C/G |  | 0.11 | 0.16 | G | **3.7E-06** | 0.65 (0.55-0.78) |  |  | 0.40 |  | 0.06 |  |  | 0.46 |  | 0.08 |
| rs2722769 | 11 | 11184950 | C/G |  | 0.07 | 0.09 | G | **0.022** | 0.77 (0.62-0.96) |  |  | 0.13 |  | 0.01 |  |  | 0.15 |  | 0.01 |
| rs7107217 | 11 | 128978900 | C/A |  | 0.44 | 0.48 | A | **0.016** | 0.86 (0.76-0.97) |  |  | 0.14 |  | 0.01 |  |  | 0.16 |  | 0.01 |
