# Supplementary material for: A Genome-Wide Association Search for Type 2 Diabetes Genes in African Americans
Source: PLoS One. 2012 Jan 4;7(1):e29202. doi: 10.1371/journal.pone.0029202 (PMC3251563; doi:10.1371/journal.pone.0029202)
Supplement: Table S10 — P-values for putative ESRD loci across the genome. SNPs selected from the GWAS (P<0.001) and associated in the Replication cohort (P<0.05 and directionally consistent) but which were not associated in the Validation cohort (P>0.05) and could represent putative ESRD loci. SNPs are ordered by chromosome and position (NCBI Build 36.1) with the major/minor alleles (positive strand) and corresponding gene (underlined) or nearest annotated gene. For each phase of the study, GWAS + Replication, Validation and Overall analyses, the additive P-value and odds ratio (OR) with associated 95% confidence interval (CI) with respect to the minor allele is listed. (DOC) [file pone.0029202.s012.doc]

**Supplementary Table 10. *P-values* for putative ESRD loci across the genome.** SNPs selected from the GWAS (P<0.001) and associated in the Replication cohort (P<0.05 and directionally consistent) but which were not associated in the Validation cohort (P>0.05) and could represent putative ESRD loci. SNPs are ordered by chromosome and position (NCBI Build 36.1) with the major/minor alleles (positive strand) and corresponding gene (underlined) or nearest annotated gene. For each phase of the study, GWAS + Replication, Validation and Overall analyses, the additive *P-value* and odds ratio (OR) with associated 95% confidence interval (CI) with respect to the minor allele is listed.

| **Locus** | | | | | **GWAS + Replication** | | **Validation** | | **Overall** | |
| --- | --- | --- | --- | --- | --- | --- | --- | --- | --- | --- |
|  |  |  |  |  | **T2DM-ESRD (n=1674)** | | **T2DM (n=1458)** | | **T2DM-ESRD + T2DM (n=3132)** | |
|  |  |  |  |  | **Control (n=1719)** | | **Control (n=1598)** | | **Control (n=3317)** | |
| **SNP** | **Chr** | **Position** | **Nearest Gene(s)** | **Alleles** | **Additive P-Value** | **OR (95% CI)** | **Additive P-Value** | **OR (95% CI)** | **Additive P-Value** | **OR (95% CI)** |
| rs11118918 | 1 | 206162312 | *PLXNA2/CD34* | G/T | **2.6E-05** | 1.26 (1.13-1.41) | 0.48 | 0.95 (0.84-1.06) | **0.010** | 1.09 (1.00-1.18) |
| rs17014015 | 1 | 207304075 | *LOC642587/-* | C/T | **2.6E-05** | 1.41 (1.20-1.66) | 0.60 | 1.03 (0.87-1.23) | **6.4E-04** | 1.20 (1.06-1.35) |
| rs1316429 | 1 | 236068519 | *LOC100130331/RYR2* | C/G | **3.6E-05** | 0.77 (0.67-0.87) | 0.66 | 1.01 (0.88-1.16) | **0.0068** | 0.88 (0.80-0.97) |
| rs10888287 | 1 | 246184040 | *OR2L13* | T/G | **8.2E-05** | 0.83 (0.75-0.91) | 0.94 | 1.01 (0.91-1.12) | **0.0034** | 0.92 (0.85-0.98) |
| rs9798973 | 3 | 164227508 |  | C/T | **3.4E-05** | 0.81 (0.73-0.90) | 0.37 | 0.94 (0.84-1.05) | **2.8E-04** | 0.88 (0.81-0.94) |
| rs4260465 | 3 | 164231047 |  | C/G | **5.5E-05** | 0.82 (0.74-0.90) | 0.54 | 0.99 (0.89-1.10) | **0.012** | 0.90 (0.84-0.97) |
| rs6838250 | 4 | 7024086 | *TBC1D14* | G/C | **6.8E-06** | 0.75 (0.66-0.85) | 0.41 | 1.04 (0.91-1.19) | **0.0066** | 0.89 (0.81-0.97) |
| rs3775043 | 4 | 96347703 | *UNC5C* | G/T | **2.0E-06** | 1.28 (1.16-1.42) | 0.77 | 1.02 (0.91-1.15) | **2.6E-04** | 1.14 (1.06-1.23) |
| rs11730446 | 4 | 96382268 | *UNC5C* | A/G | **9.4E-05** | 1.21 (1.09-1.34) | 0.59 | 0.97 (0.88-1.08) | **0.013** | 1.08 (1.00-1.16) |
| rs891382 | 4 | 147371791 | *SLC10A7/LSM6* | A/G | **1.9E-05** | 1.49 (1.24-1.81) | 0.93 | 0.98 (0.81-1.18) | **0.0022** | 1.20 (1.05-1.37) |
| rs247096 | 5 | 128121777 | *SLC27A6/FBN2* | T/C | **1.4E-06** | 1.33 (1.18-1.49) | 0.43 | 0.98 (0.87-1.11) | **0.0029** | 1.14 (1.04-1.24) |
| rs2161305 | 5 | 128133407 | *SLC27A6/FBN2* | T/A | **1.4E-06** | 1.32 (1.18-1.49) | 0.43 | 0.97 (0.86-1.09) | **0.0029** | 1.13 (1.04-1.23) |
| rs3822908 | 6 | 97706334 | *C6orf167* | T/C | **5.8E-05** | 0.76 (0.66-0.87) | 0.55 | 1.11 (0.96-1.28) | **0.012** | 0.92 (0.83-1.02) |
| rs2008860 | 6 | 108418961 | *OSTM1/SEC63* | C/G | **3.5E-05** | 1.27 (1.13-1.43) | 0.94 | 1.02 (0.90-1.16) | **0.0021** | 1.14 (1.04-1.24) |
| rs208865 | 6 | 130114250 | *C6orf191/ARHGAP18* | C/T | **2.9E-05** | 1.41 (1.19-1.65) | 0.86 | 0.98 (0.82-1.18) | **0.0016** | 1.17 (1.03-1.32) |
| rs1467404 | 6 | 133131835 | *C6orf192* | T/C | **5.5E-05** | 0.82 (0.74-0.90) | 0.66 | 1.00 (0.90-1.11) | **0.0085** | 0.91 (0.84-0.98) |
| rs9493454 | 6 | 133186323 | *LOC285735/RPS12* | A/C | **1.3E-05** | 1.25 (1.13-1.38) | 0.42 | 0.98 (0.88-1.09) | **0.0090** | 1.10 (1.02-1.18) |
| rs7769051 | 6 | 133188490 | *LOC285735/RPS12* | C/A | **1.7E-06** | 1.29 (1.16-1.43) | 0.77 | 1.07 (0.95-1.20) | **2.2E-04** | 1.17 (1.08-1.26) |
| rs6963635 | 7 | 83431742 | *SEMA3A* | C/T | **7.0E-05** | 0.55 (0.41-0.74) | 0.64 | 0.90 (0.67-1.20) | **0.0013** | 0.71 (0.57-0.87) |
| rs773506 | 9 | 93015293 | *AUH/SYK* | G/A | **1.4E-05** | 0.76 (0.67-0.86) | 0.95 | 1.00 (0.89-1.13) | **0.0018** | 0.88 (0.80-0.96) |
| rs2904532 | 12 | 65582312 | *CAND1/GRIP1* | A/G | **4.2E-05** | 0.76 (0.67-0.87) | 0.80 | 0.98 (0.86-1.12) | **0.0049** | 0.87 (0.79-0.95) |
| rs11176482 | 12 | 65582815 | *CAND1/GRIP1* | T/C | **4.3E-05** | 0.76 (0.66-0.87) | 0.87 | 0.98 (0.85-1.12) | **0.0041** | 0.86 (0.79-0.95) |
| rs12302041 | 12 | 76654357 | *NAV3/-* | G/A | **1.5E-05** | 1.53 (1.26-1.85) | 0.88 | 0.99 (0.80-1.23) | **0.0022** | 1.22 (1.06-1.41) |
| rs9533481 | 13 | 42887186 | *ENOX1* | T/C | **9.1E-07** | 1.28 (1.16-1.42) | 1.00 | 1.01 (0.90-1.12) | **3.5E-04** | 1.13 (1.05-1.22) |
| rs1950321 | 14 | 39076936 | *-/FBXO33* | C/G | **2.6E-05** | 1.50 (1.23-1.82) | 0.68 | 0.94 (0.75-1.18) | **8.3E-04** | 1.18 (1.01-1.37) |
| rs1504608 | 14 | 56542042 | *EXOC5/OTX2OS1* | T/C | **4.4E-05** | 0.81 (0.73-0.89) | 0.86 | 1.03 (0.92-1.16) | **0.0020** | 0.92 (0.85-0.99) |
| rs1978243 | 14 | 86596161 |  | C/T | **2.4E-50** | 0.78 (0.70-0.88) | 0.57 | 1.03 (0.92-1.17) | **0.0072** | 0.90 (0.83-0.98) |
| rs6494387 | 15 | 61132580 | *TPM1* | G/A | **1.6E-05** | 1.25 (1.13-1.39) | 0.86 | 0.96 (0.86-1.07) | **0.0025** | 1.09 (1.01-1.18) |
| rs372889 | 19 | 18034604 | *IL12RB1* | C/T | **7.9E-06** | 1.24 (1.13-1.36) | 0.78 | 1.05 (0.94-1.17) | **6.1E-04** | 1.13 (1.06-1.22) |
| rs9981433 | 21 | 40231436 | *DSCAM/PCP4* | T/G | **7.1E-07** | 0.69 (0.59-0.80) | 0.68 | 1.03 (0.88-1.21) | **9.1E-04** | 0.85 (0.76-0.95) |
| rs1034589 | 22 | 29909234 | *RNF185* | T/C | **1.6E-05** | 0.60 (0.47-0.75) | 1.00 | 0.91 (0.72-1.15) | **0.0017** | 0.74 (0.63-0.87) |
| rs2106294 | 22 | 29975760 | *LIMK2* | T/C | **6.6E-06** | 0.58 (0.45-0.73) | 0.81 | 0.87 (0.69-1.10) | **5.8E-04** | 0.71 (0.60-0.84) |
| rs4820043 | 22 | 29977095 | *LIMK2* | G/A | **8.2E-06** | 0.58 (0.46-0.74) | 0.99 | 0.90 (0.72-1.14) | **0.0012** | 0.73 (0.62-0.86) |
| rs5749682 | 22 | 32578315 | *LARGE* | C/A | **7.9E-05** | 0.65 (0.53-0.81) | 0.54 | 1.07 (0.85-1.35) | **0.014** | 0.84 (0.72-0.98) |
| rs735853 | 22 | 35009162 | *MYH9* | C/G | **1.3E-05** | 0.69 (0.59-0.82) | 0.69 | 0.94 (0.80-1.11) | **5.4E-04** | 0.81 (0.72-0.91) |
